# Activity-dependent modulation of neuronal K_V_ channels by retinoic acid enhances Ca_V_ channel activity

**DOI:** 10.1016/j.jbc.2022.101959

**Published:** 2022-04-20

**Authors:** Eric de Hoog, Gaynor E. Spencer

**Affiliations:** Department of Biological Sciences, Brock University, St. Catharines, Ontario, Canada

**Keywords:** retinoic acid, L-type channels, non–L-type channels, voltage clamp, voltage-gated K channels, A-type channels, delayed rectifier channels, RAR, RXR, *Lymnaea stagnalis*, 4-AP, 4-aminopyridine, AHP, afterhyperpolarization, Ca_V_, voltage-gated Ca^2+^ channel, CNS, central nervous system, DM, defined medium, DMSO, dimethyl sulfoxide, I_A_, A-type channel current, I_KD_, delayed rectifier current, IV, current–voltage, K_V_, voltage-gated K^+^ channel, LTD, long-term depression, LTP, long-term potentiation, RA, retinoic acid, RMP, resting membrane potential, RPA, right parietal A, RPB, right parietal B, RPeD1, right pedal dorsal 1, TEA, tetraethyl ammonium, VF, visceral F

## Abstract

The metabolite of vitamin A, retinoic acid (RA), is known to affect synaptic plasticity in the nervous system and to play an important role in learning and memory. A ubiquitous mechanism by which neuronal plasticity develops in the nervous system is through modulation of voltage-gated Ca^2+^ (Ca_V_) and voltage-gated K^+^ channels. However, how retinoids might regulate the activity of these channels has not been determined. Here, we show that RA modulates neuronal firing by inducing spike broadening and complex spiking in a dose-dependent manner in peptidergic and dopaminergic cell types. Using patch-clamp electrophysiology, we show that RA-induced complex spiking is activity dependent and involves enhanced inactivation of delayed rectifier voltage-gated K^+^ channels. The prolonged depolarizations observed during RA-modulated spiking lead to an increase in Ca^2+^ influx through Ca_V_ channels, though we also show an opposing effect of RA on the same neurons to inhibit Ca^2+^ influx. At physiological levels of Ca^2+^, this inhibition is specific to Ca_V_2 (not Ca_V_1) channels. Examining the interaction between the spike-modulating effects of RA and its inhibition of Ca_V_ channels, we found that inhibition of Ca_V_2 channels limits the Ca^2+^ influx resulting from spike modulation. Our data thus provide novel evidence to suggest that retinoid signaling affects both delayed rectifier K^+^ channels and Ca_V_ channels to fine-tune Ca^2+^ influx through Ca_V_2 channels. As these channels play important roles in synaptic function, we propose that these modulatory effects of retinoids likely contribute to synaptic plasticity in the nervous system.

Neural circuits are able to process and store information largely as a result of their ability to undergo plasticity and modulation. A ubiquitous mechanism through which neuronal plasticity occurs is through modulation of voltage-gated Ca^2+^ (Ca_V_) and voltage-gated K^+^ (K_V_) channels (1, 2). Ca_V_ channel activity influences synaptic transmission and gene transcription, whereas K_V_ channel activity regulates action potential repolarization and neuronal excitability. Neurons can possess three major subtypes of Ca_V_ channels, Ca_V_1, Ca_V_2, and Ca_V_3. The Ca_V_1 (L-type) channels are activated at high voltages, are largely present in neuronal dendrites and the cell body, and mediate activity-dependent gene transcription (3, 4). Ca_V_2 (non–L-type) channels also activate at high voltages, are largely present at presynaptic terminals and mediate synaptic transmission, though can also mediate Ca^2+^ signaling in neuronal cell bodies (1, 4). Finally, Ca_V_3 (T-type) channels activate at low voltages and shape the firing properties of neurons (5). There are many different gene families for K_V_ channels. Two broad categories of K_v_ channels are the rapidly inactivating A-type channels and the nonrapidly inactivating delayed rectifier channels (6). Both these categories of K_V_ channels have been implicated in shaping the firing properties of neurons, (*e.g.*, hippocampal neurons) and have thus been implicated in synaptic plasticity in the nervous system (2, 7).

Retinoic acid (RA), the active metabolite of vitamin A, has recently emerged as a critical regulator of neural plasticity in the adult brain (in addition to its important roles in nervous system development). It can exert both genomic and nongenomic effects by binding to retinoic acid receptors and retinoid X receptors (RXR) (8, 9), though its ability to bind directly to other signaling molecules has also been shown (10). RA signaling plays an important role in spatial learning and memory in rodents (11, 12, 13), and at the cellular level, it contributes to long-term potentiation (LTP) and/or long-term depression (LTD) in the hippocampus (11, 13, 14). RA signaling can also interact with Ca^2+^ signaling during homeostatic plasticity at excitatory synapses (8, 15, 16, 17). In particular, activation of RA signaling induces local synthesis and membrane insertion of glutamate receptors, subsequently increasing Ca^2+^ influx into the postsynaptic neurons in an activity-dependent manner (8, 15, 16, 17). RA has also been shown to reduce inhibitory synaptic transmission through rapid endocytosis of postsynaptic gamma-aminobutyric acid type A receptors (18). In addition to these postsynaptic effects of RA, it can both increase or decrease inhibitory synaptic transmission through presynaptic mechanisms, in a cell type–specific manner (19, 20). Thus, RA signaling can regulate activity-dependent plasticity at different synapses through diverse presynpatic and postsynaptic mechanisms.

In addition to modulating synaptic efficacy in the vertebrate central nervous system (CNS), RA signaling also affects neural circuitry in invertebrates. We have previously shown that RA is required for normal associative learning and memory in the mollusc, *Lymnaea stagnalis* (21), and can affect neuronal synapse formation and modulation *in vitro* (22). However, the activity-dependent mechanisms by which RA regulates neural plasticity are largely unknown. In *Lymnaea* neurons, RA produces spike broadening and complex spiking, which includes burst firing and plateau potentials (23). Such a modulation of spike shape and firing patterns is known to play a role in synaptic plasticity (during learning and memory) and can result in enhanced synaptic transmission in neural circuits (24). We have also shown that RA signaling inhibits Ca_V_ channels in *Lymnaea* neurons (25). Whether the RA-induced spike broadening and complex spiking is activity dependent (and thus contributes to activity-dependent processes such as learning and memory), and how this effect interacts with concurrent RA-mediated inhibition of Ca_V_ channels, is not yet known. In this study, we examine this interaction and also determine whether changes in K_V_ channel function contribute to these modulatory effects of RA on spike broadening and complex spiking activity.

We show that micromolar concentrations of RA (similar to those eliciting effects in mouse and human neurons) produce spike broadening and complex spiking in an activity-dependent manner and in diverse neuronal cell types. By playing back neuronal firing patterns (action potential clamp) into single neurons *in vitro*, we show that RA-mediated inhibition of Ca_V_2 channels limits the enhanced Ca^2+^ influx that occurs during RA-mediated spike broadening and activity-dependent complex spiking. We also determine that spike broadening and activity-dependent complex spiking likely occurs as a result of RA-mediated inhibition of delayed rectifier K_V_ channels. These data provide novel insights into how multiple forms of neuromodulation mediated by RA might tune Ca^2+^ influx (and thus potentially neurotransmitter release) at neuronal synapses.

## Results

### RA-induced neuromodulation is both concentration and activity dependent

Spike broadening and burst firing are important for information transfer and plasticity in neural circuits. We have previously shown that all-*trans* RA produces spike broadening and complex spiking (burst firing and plateau potentials) in *Lymnaea* visceral F (VF) neurons (23). However, the activitydependence of these effects, as well as the underlying cellular mechanisms, has not yet been determined.

**Figure 1:**
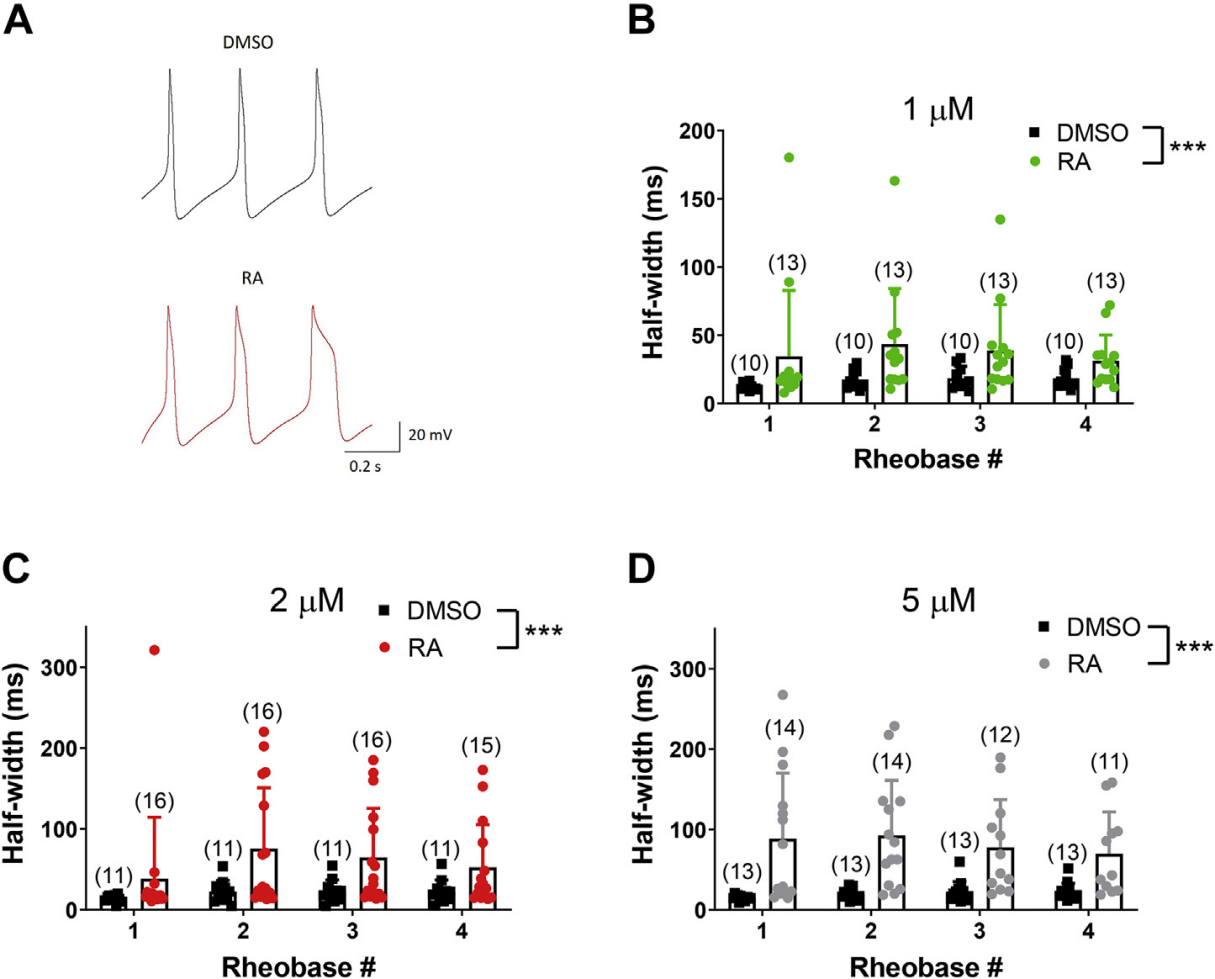
**RA produces spike broadening at low micromolar concentrations.***A*, the first three action potentials produced in response to current injection were recorded in the presence of either 0.01% DMSO or 1 μM RA. Representative traces illustrate spike broadening in RA (*red*) compared with DMSO (*black*). *B*–*D*, graphs illustrating that RA significantly increased the mean half-width (in milliseconds) of VF cell action potentials, compared with DMSO, indicating RA-induced spike broadening. Data obtained with 1 μM RA (*B*; *green*), 2 μM RA (*C*; *red*), and 5 μM RA (*D*; *gray*) are shown, though similar significant results were also obtained with 2.5 and 3 μM RA. ∗∗∗*p* ≤ 0.001. Error bars represent SD. Bracketed values over bars represent n values. Rheobase 1 indicates the first current step that elicited a minimum of three action potentials, and rheobases 2 to 4 were the three subsequent depolarizing current injection steps. No significant effects of rheobase were found, and so the effects of RA on spike broadening were not activity dependent. DMSO, dimethyl sulfoxide; RA, retinoic acid; VF, visceral F.

**Figure 2:**
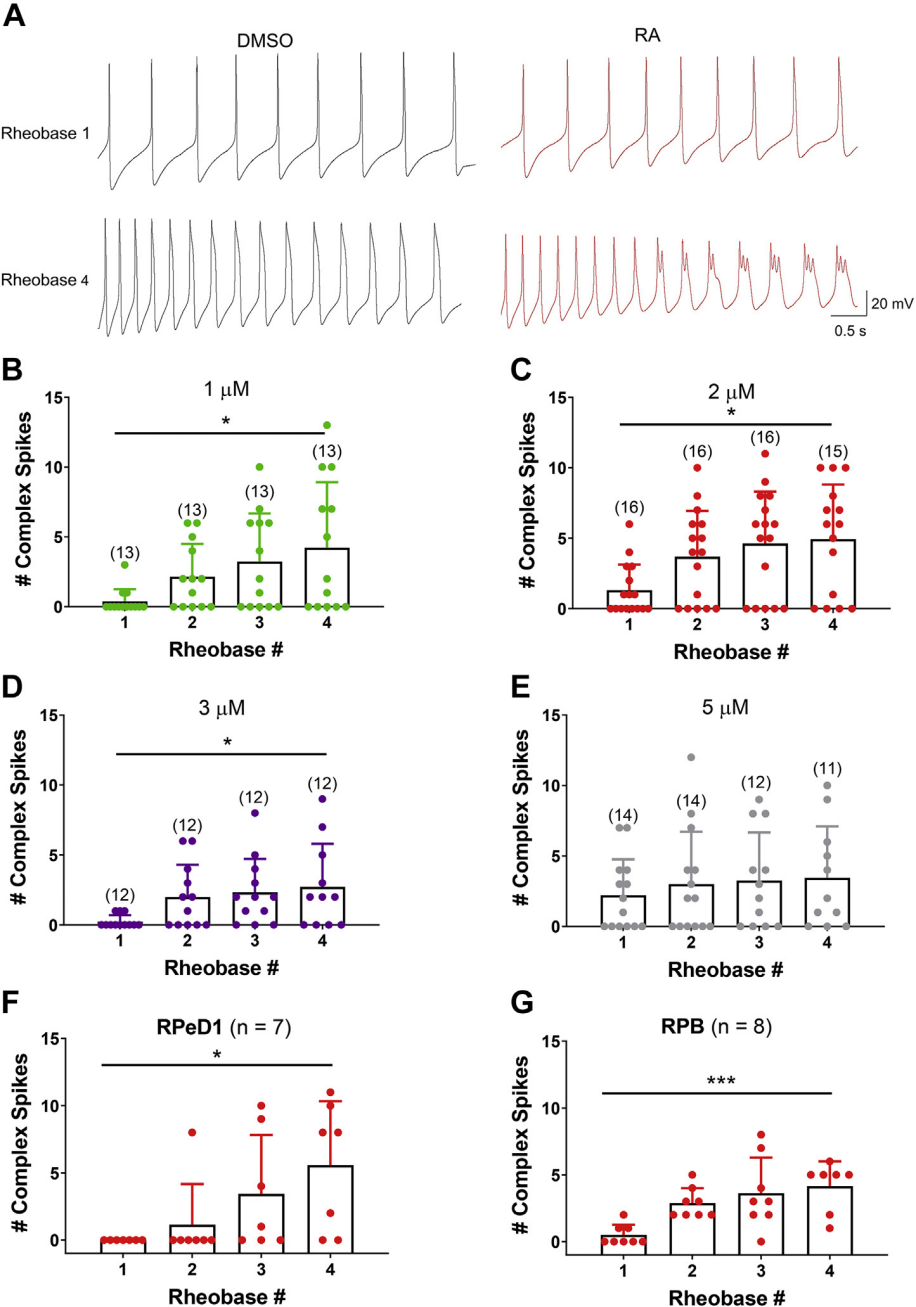
**RA produces activity-dependent complex spiking in multiple cell types.***A*, raw recordings of firing activity in VF neurons at either rheobase 1 (*top*) or rheobase 4 (*bottom*) in the presence of either 0.01% DMSO (*black*) or 1 μM RA (*red*). Representative traces illustrate the significant increase in spikes elicited from rheobase 1 to 4 as well as the presence of complex spikes in the presence of RA (but not DMSO) at rheobase 4. *B*–*E*, graphs illustrating the rheobase/activity-dependent increase in complex spiking in VF cells induced by 1 μM RA (*B*; *green*), 2 μM RA (*C*; *red*), and 3 μM atRA (*D*; *purple*), but not by 5 μM RA (*E*; *gray*). ∗*p* ≤ 0.05. Bracketed values over bars represent n values. *F* and *G*, a significant activity-dependent increase in complex spiking was also observed in the dopaminergic (RPeD1) cell type (*F*) and the neuroendocrine right parietal B (RPB) cells (*G*) following exposure to 2 μM RA. ∗*p* ≤ 0.05; ∗∗∗*p* ≤ 0.001. Error bars represent SD. atRA, all-*trans* retinoic acid; DMSO, dimethyl sulfoxide; RA, retinoic acid; RPeD1, right pedal dorsal 1; VF, visceral F.

VF neurons were incubated overnight in varying concentrations of RA (ranging from 1 to 5 μM) or the equivalent concentration of dimethyl sulfoxide (DMSO) (as controls). The following day, individual neurons were current clamped and stimulated using a current-step protocol ranging from −100 to 400 pA in intervals of 25 pA. To determine whether RA-mediated spike broadening and complex spiking was activity dependent, we analyzed spike half-width (spike broadening) and complex spiking at the first current step that elicited a minimum of three action potentials (rheobase 1) and at the three subsequent depolarizing current injection steps (rheobases 2–4). We found that all concentrations of RA between 1 and 5 μM produced spike broadening (representative example is shown in Fig. 1*A*). We compared the spike half-width in the presence or the absence of RA at all four rheobases and for each concentration of RA. A two-way ANOVA of spike half-width at each concentration (1, 2, 2.5, 3, and 5 μM RA) revealed a significant effect of treatment (RA), but not rheobase (activity), for all concentrations including the lowest concentrations of 1 μM (*F*_(1,84)_ = 11.546; *p* = 0.001; Fig. 1*B*) and 2 μM (*F*_(1,99)_ = 13.173; *p* < 0.001; Fig. 1*C*).

We also determined whether RA influenced other physiological properties of neuronal activity. A two-way ANOVA of the peak voltage of the action potential across rheobases 1 to 4, for each concentration of RA, revealed that all concentrations of RA (except 1 μM) significantly reduced the action potential peak voltage, including the lower concentration of 2 μM (*F*_(1,99)_ = 5.175; *p* = 0.025; RA: 39.24 ± 9.7 mV; DMSO: 41.9 ± 5.7 mV). Similarly, all concentrations of RA (including the lowest concentration of 1 μM [*F*_(1,84)_ = 74.152; *p* < 0.001]) significantly depolarized the peak voltage of the afterhyperpolarization (AHP) (1 μM RA: −48.93 ± 6.4 mV; DMSO: −58.91 ± 5.8 mV), indicative of a reduced AHP amplitude. However, there were minimal effects of RA on the resting membrane potential (RMP) or input resistance; only 2.5 μM RA had a significant effect on the RMP (RA: −52.77 ± 7.1 mV; DMSO: −44.25 ± 7.7 mV; *t* = −2.572; *p* = 0.019), whereas only 3 μM significantly reduced the input resistance (RA: 661.24 ± 275.5 MΩ; DMSO: 919.86 ± 273.2 MΩ; *t* = −2.137; *p* = 0.046).

To determine the concentration dependence of complex spiking induced by RA, statistical analysis (Fisher's exact tests) revealed that all concentrations of RA produced a significant increase in the proportion of cells exhibiting complex spiking (1 μM RA: 69%; *p* = 0.002; 2 μM RA: 81%; *p* < 0.001; 2.5 μM RA: 82%; *p* < 0.001; 3 μM RA: 75%; *p* = 0.001; 5 μM RA: 86%; *p* < 0.001) compared with the equivalent concentrations of DMSO, at which no cells (0%) exhibited complex spiking. These data suggest that concentrations of RA in a range similar to those previously estimated to occur in the *Lymnaea* CNS (26), and similar to those that induce effects in vertebrate neurons, produce both spike broadening, changes in spike amplitude and AHP, as well as complex firing in individual VF neurons.

To determine whether the effect of RA on complex spiking was activity dependent, we compared the number of complex spikes that occurred across rheobases 1 to 4, for each RA concentration. An example of complex spikes in the presence of 1 μM RA (at rheobase 4) is shown in Figure 2*A*. These representative recordings in Figure 2*A* also illustrate how the number of elicited spikes significantly increased from rheobase 1 to 4 (1 μM RA: H = 8.827; *p* = 0.032, ANOVA on ranks). This was also true for 2, 3, and 5 μM RA (and DMSO), confirming an activity-dependent increase in spike number across rheobases. A one-way ANOVA on ranks revealed that the incidence of complex spiking was found to be activity dependent at lower concentrations of 1 μM RA (H = 8.177; *p* = 0.042; Fig. 2*B*), 2 μM RA (H = 9.019; *p* = 0.029; Fig. 2*C*), and 3 μM RA (H = 9.138; *p* = 0.028; Fig. 2*D*), but not at the higher concentration of 5 μM (H = 1.003; *p* = 0.8; Fig. 2*E*). A concentration of 2 μM RA, which elicits both spike broadening and activity-dependent complex spiking, was chosen for use throughout the remainder of this study.

### RA-induced neuromodulation occurs across multiple cell types

We next determined whether RA would produce spike broadening and activity-dependent complex spiking in other neuronal cell types, or whether this was specific to VF neurons. We assessed the effects of RA on the large dopaminergic interneuron, right pedal dorsal 1 (RPeD1, n = 7), the neuroendocrine right parietal B (RPB) cells (n = 8), or the right parietal A (RPA) respiratory motor neurons (n = 6). A two-way ANOVA of spike half-width revealed a significant effect of treatment on RPeD1 (*F*_(1,48)_ = 61.375; *p* < 0.001), RPB (*F*_(1,54)_ = 12.363; *p* < 0.001), and RPA cells (*F*_(1,40)_ = 16.601; *p* < 0.001), indicating that RA significantly increased spike half-width in all three cell types (compared with DMSO). RA also significantly increased the proportion of cells exhibiting complex spiking at any of the four rheobases (RPeD1: RA 71.4%, DMSO 0%; *p* = 0.021; RPB: RA 100%, DMSO 0%; *p* < 0.001; RPA: RA 83.3%, DMSO 0%; *p* = 0.015; Fisher’s exact tests). The ability of RA to induce complex spiking was also activity dependent in RPeD1 (H = 9.998; *p* = 0.019; Fig. 2*F*) and RPB cells (H = 16.395; *p* < 0.001; Fig. 2*G*) but not in RPA cells (*F*_(3,20)_ = 1.114; *p* = 0.367). These data suggest that RA induces activity-dependent complex spiking in diverse neuronal subtypes, including dopaminergic and peptidergic cells, suggesting a possible wide range of influence on distinct cell types within the brain.

### RA modulates Ca_V_2 channels in physiological [Ca^2+^]

As Ca_V_ channels play an ubiquitous role in synaptic transmission and activity-dependent changes in gene expression, modulation of their activity might directly influence activity-dependent changes in neural circuits. We have previously shown that RA (1 and 5 μM) inhibits Ca_V_ channels by shifting the voltage dependence of channel activation; a biophysical property that determines at which voltage the channels open, thus determining their activity during neuronal firing. However, the previous study was performed using 10 mM barium as the charge carrier, eliminating any subsequent effects of calcium influx (25). As RA is known to interact with Ca^2+^ signaling at hippocampal synapses (17, 27), we deemed it necessary to examine the effects of RA on Ca_V_ channels in the presence of physiological concentrations of extracellular Ca^2+^.

**Figure 3:**
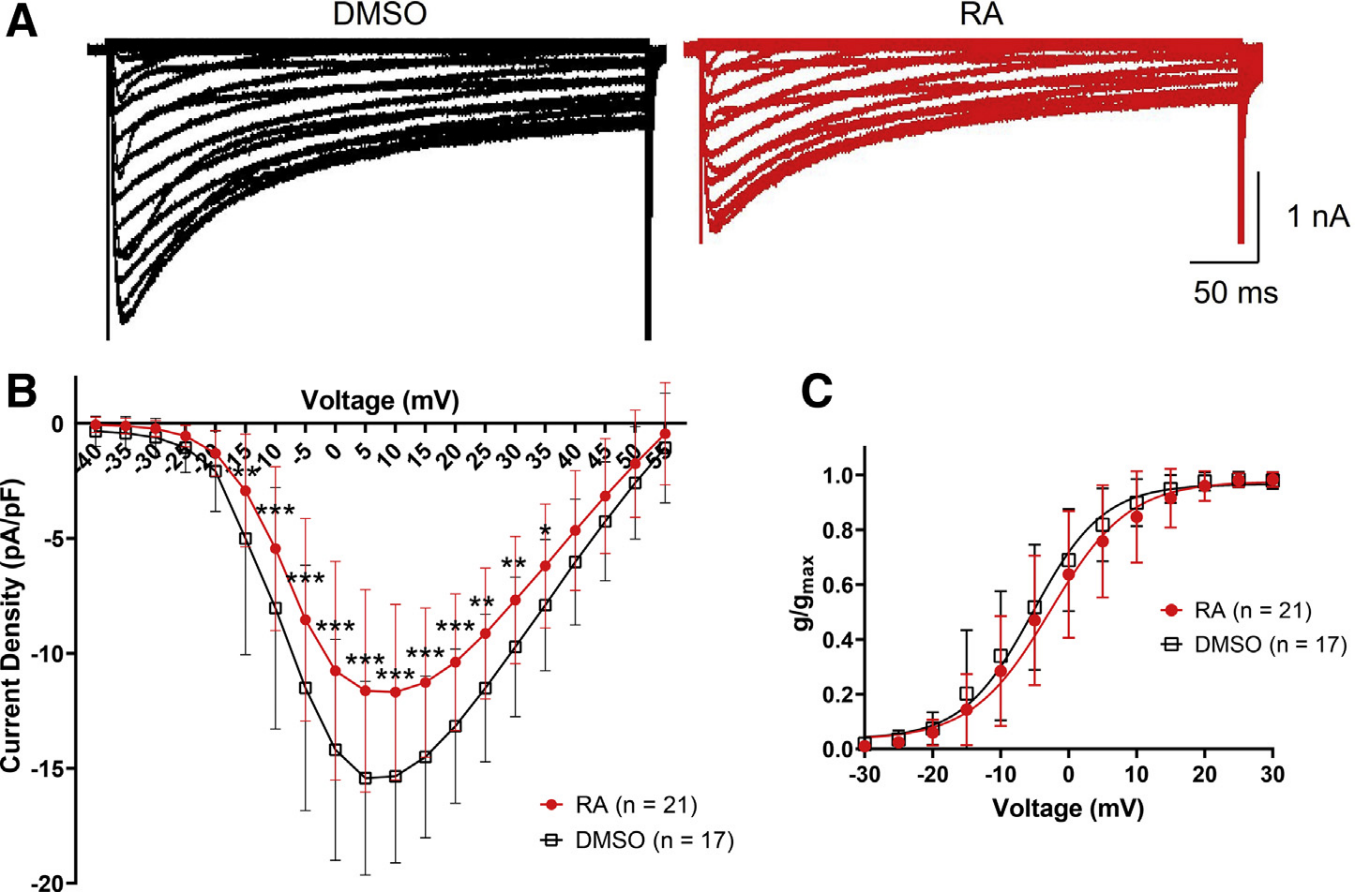
**RA inhibits I**_**Ca**_**in physiological levels of Ca**^**2+**^**.***A*, raw representative recordings of I_Ca_ in VF neurons following exposure to either 0.02% DMSO (*black traces*, *left*) or 2 μM RA (*red traces*, *right*), illustrating the reduced peak I_Ca_ in the presence of RA. *B*, IV relationship showing that RA (*red circles*) inhibited peak current density (I_Ca_) at voltages between −15 and +35 mV. ∗*p* ≤ 0.05; ∗∗*p* ≤ 0.01; ∗∗∗*p* ≤ 0.001 (compared with DMSO, *black squares*). *C*, activation curve illustrating that RA (*red circles*) had no significant effect on the voltage dependence of channel activation, compared with DMSO (*black squares*). Error bars represent SD. DMSO, dimethyl sulfoxide; I_Ca_, calcium current; IV, current–voltage; RA, retinoic acid; VF, visceral F.

VF neurons were cultured overnight in the presence or the absence of RA and then voltage clamped in the presence of extracellular Ca^2+^ (4.1 mM). Cells were held at −115 mV for 1 s and then stepped to potentials between −115 mV and +55 mV for 400 ms, in 5 mV increments. Raw recordings in Figure 3*A* illustrate the reduction in I_Ca_ in the presence of 2 μM RA. A two-way ANOVA of peak current density revealed a significant interaction between treatment and voltage (*F*_(34,1260)_ = 2.758; *p* < 0.001). The current–voltage (IV) relationship in Figure 3*B* illustrates that RA inhibited I_Ca_ at potentials between −15 and +35 mV. However, RA did not significantly affect the voltage dependence of channel activation (Fig. 3*C*), the voltage of half-maximal activation (RA: −2.683 ± 7.1 mV; DMSO: −5.224 ± 6.4 mV; *p* = 0.260), or the slope factor (RA: 5.777 ± 1.9; DMSO: 5.179 ± 1.6; *p* = 0.186).

Two additional properties that influence Ca^2+^ influx through Ca_V_ channels are the voltage dependence of inactivation (the inactivation that occurs at a particular membrane potential) and the recovery from inactivation (the time dependence of the removal of inactivation at negative membrane potentials). However, a two-way ANOVA of I_Ca_ inactivation revealed that treatment (RA *versus* DMSO) had no effect on the voltage dependence of inactivation (*F*_(1,490)_ = 1.252; *p* = 0.264). RA had no effect on the voltage of half-maximal inactivation (RA: −14.362 ± 4.5 mV; DMSO: −12.091 ± 4.4 mV; *t* = −1.021; *p* = 0.324) or the slope of inactivation (RA: 4.88 ± 0.4; DMSO: 4.504 ± 0.7; *t* = 1.313; *p* = 0.210). A two-way ANOVA of recovery from inactivation also revealed no significant effect of treatment (*F*_(1,294)_ = 0.242; *p* = 0.623), suggesting that RA had no effect on the recovery from inactivation of Ca_V_ channels. Though it was not possible to record firing activity and Ca^2+^ currents in the same cell (because of the presence of K^+^ channel blockers), overall these data indicate that concentrations of RA that produced activity-dependent complex spiking also inhibited I_Ca_ in the same cell type.

### RA specifically inhibits Ca_V_2 channels in physiological [Ca^2+^]

We next determined, using physiological concentrations of Ca^2+^, whether RA specifically affected L-type or non–L-type channels. VF neurons were cultured in the presence or the absence of RA overnight and then voltage clamped to record I_Ca_. The Ca_V_1 (L-type) channel blocker, nifedipine (Sigma–Aldrich; 10 μM), was utilized to separate Ca_V_2 (non–L-type) currents from Ca_V_1 (L-type) currents (28, 29). The proportional block of I_Ca_ by nifedipine indicated that Ca_V_1 current comprised approximately 20 to 25% of total I_Ca_. The proportional block by nifedipine was slightly (but nonsignificantly) greater following RA treatment, compared with DMSO (RA: 26.839% ± 15.7; DMSO: 20.26% ± 12.1; *t* = 1.008; *p* = 0.329).

**Figure 4:**
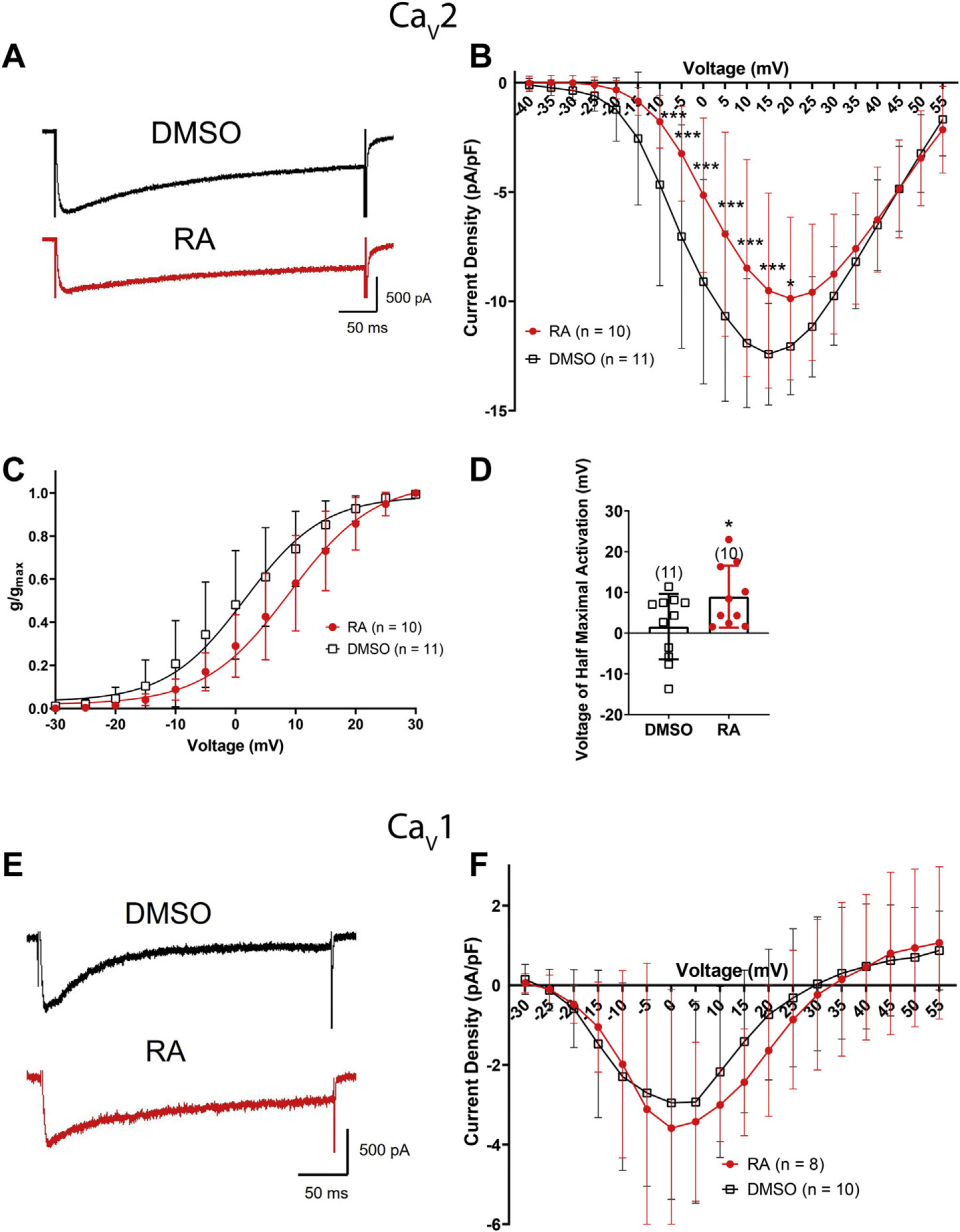
**RA inhibits Ca**_**V**_**2 channels by shifting the voltage dependence of channel activation.***A*, raw representative recordings (obtained at +5 mV) illustrating the reduced amplitude of I_Ca_ through Ca_v_2 channels following exposure to 2 μM RA (*red*, *lower trace*) compared with 0.02% DMSO (*black*, *upper trace*). *B*, IV relationship showing that 2 μM RA (*red circles*) inhibited the peak current density through Ca_V_2 channels at potentials from −10 to +20 mV. *C*, activation curve illustrating that RA shifted the voltage dependence of channel activation of Ca_V_2 channels to more positive potentials. *D*, RA significantly increased the voltage of half-maximal activation, compared with DMSO. *Bracketed values* represent n values. ∗*p* ≤ 0.05; ∗∗*p* ≤ 0.01; and ∗∗∗*p* ≤ 0.001 (compared with DMSO). Error bars represent SD. *E*, raw representative recordings (obtained at +5 mV) showing no difference in Ca_V_1 channel currents following exposure to either 2 μM RA (*red*, *lower trace*) or to 0.02% DMSO (*black*, *upper trace*). *F*, IV relationship showing that 2 μM RA (*red circles*) had no significant effect on peak current density of Ca_V_1 channels compared with DMSO (*black squares*). Ca_V_, voltage-gated Ca^2+^channel; DMSO, dimethyl sulfoxide; I_Ca_, calcium current; IV, current–voltage; RA, retinoic acid.

A two-way ANOVA of nifedipine-insensitive (Ca_V_2; non–L-type) I_Ca_ revealed a significant interaction between treatment (RA *versus* DMSO) and voltage (*F*_(34,665)_ = 2.244; *p* < 0.001). Raw recordings (Fig. 4*A*) and the IV relationship (Fig. 4*B*) illustrate the inhibition of Ca_V_2 channels by RA at voltages ranging from −10 to +20 mV. Furthermore, the activation curve in Figure 4*C* illustrates that RA inhibited Ca_V_2 by shifting the voltage dependence of channel activation to more positive potentials; RA significantly increased the voltage of half-maximal activation (Fig. 4*D*) but had no significant effect on slope factor (RA: 7.101 ± 1.6; DMSO: 6.533 ± 1.3; *t* = 0.906; *p* = 0.376). In contrast, RA had no significant effect on Ca_V_1 channels (Fig. 4, *E* and *F*). These data suggest that concentrations of RA (2 μM), known to produce activity-dependent complex spiking in these cells, also inhibited Ca_V_2 (but not Ca_V_1) channels, by shifting the voltage dependence of channel activation to more positive potentials.

### RA-mediated inhibition of Ca_V_ channels limits Ca^2+^ entry during activity-dependent neuromodulation

The aforementioned results indicate that the RA-mediated spike broadening and activity-dependent complex spiking occurs in the same cell type and at the same concentrations as the RA-mediated inhibition of Ca_V_2 channels. Presumably, the prolonged depolarizations occurring during spike broadening and complex spiking would enhance Ca^2+^ influx through Ca_V_ channels, and it is possible that, if occurring concurrently, RA might induce opposing effects on Ca^2+^ signaling. We next determined whether spike broadening and activity-dependent complex spiking does indeed enhance Ca^2+^ influx, and if so, whether this is influenced by concurrent RA-induced inhibition of Ca_V_2 channels. To this end, we utilized the action potential clamp technique, which uses previously recorded neuronal activity (spiking) as the voltage stimulus for recording ion channel activity. Specifically, the voltage stimulus protocol consisted of a total of nine action potentials obtained from firing activity (rheobase 4) in a cell exposed to either 2 μM RA or 0.02% DMSO. The voltage protocol from DMSO treatment will be referred to hereafter as the “control” voltage protocol, whereas that from the RA-treated cell (exhibiting spike broadening and complex spiking) will be referred to as the “modulated” voltage protocol.

**Figure 5:**
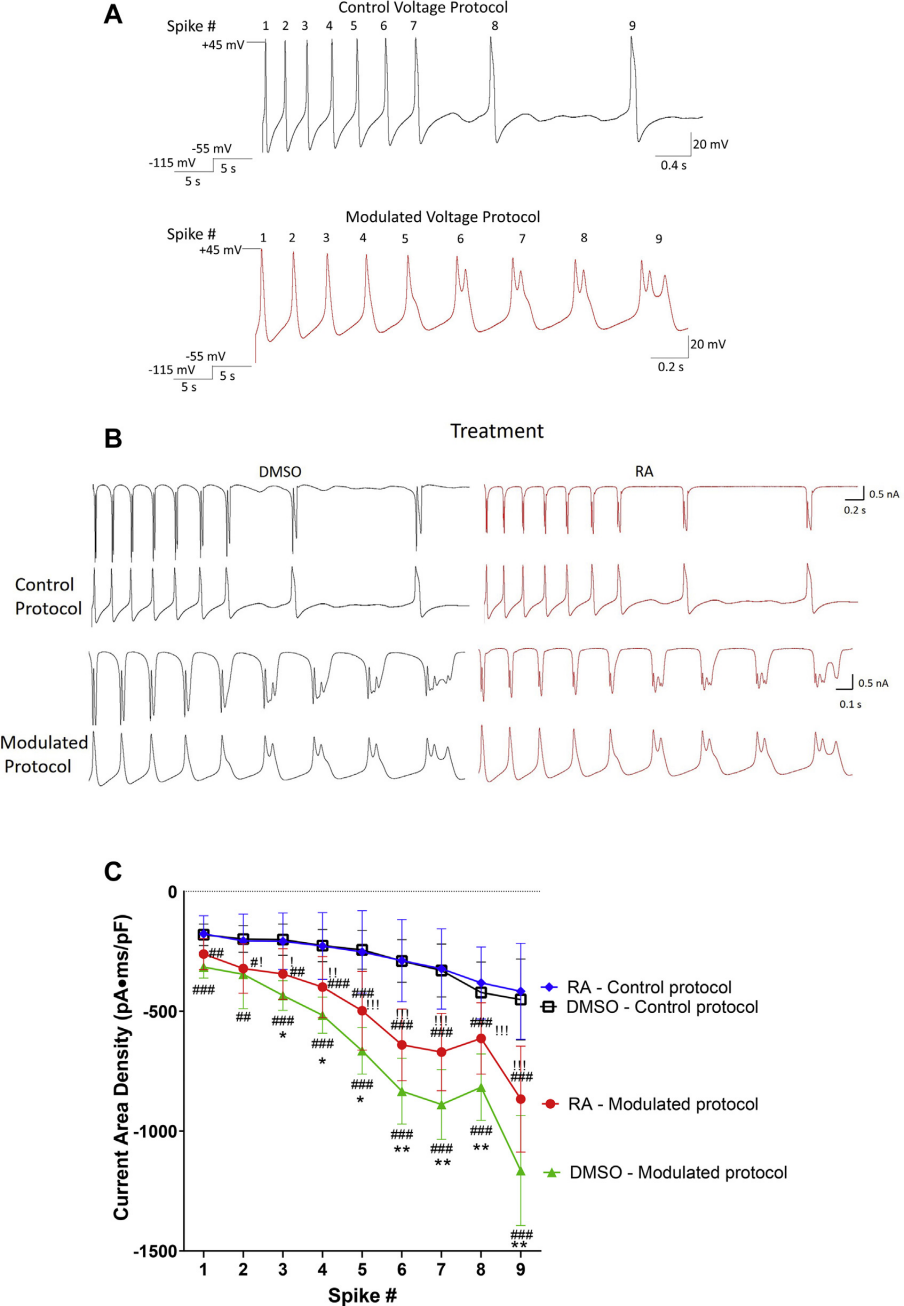
**Spike broadening and complex spiking enhances Ca**^**2+**^**influx *via* Ca**_**V**_**channels, but this is limited by concurrent exposure to RA.***A*, illustrations of the control and modulated voltage protocols. The control protocol consists of nine action potentials recorded from a DMSO-exposed cell (at rheobase 4), whereas the modulated protocol consists of nine action potentials recorded from an RA-exposed cell. *B*, raw recordings of I_Ca_ (*top traces*) corresponding to the nine action potentials comprising the control and modulated voltage protocols (*bottom traces*), in the presence of either 0.02% DMSO (*black*, *left*) or 2 μM RA (*red*, *right*). *C*, the modulated voltage protocol significantly enhanced I_Ca_ (in cells exposed to both RA [*red*, n = 11] or DMSO [*green*, n = 9]), compared with the control voltage protocols (in cells exposed to either RA [*blue*, n = 11] or DMSO [*black*, n = 9]). # represents the comparison between modulated and control protocols (^#^*p* ≤ 0.05; ^##^*p* ≤ 0.01; and ^###^*p* ≤ 0.001). The modulated protocol applied to cells exposed to DMSO showed enhanced I_Ca_ compared with the modulated protocol in cells exposed to RA. ∗ represents the comparison between modulated protocols in RA (*red circles*) and DMSO (*green triangles*) (∗*p* ≤ 0.05; ∗∗*p* ≤ 0.01). ! represents the enhanced I_Ca_ between the modulated protocol in RA (*red circles*) and the control protocol in DMSO (*black squares*) (!*p* ≤ 0.05; !!*p* ≤ 0.01; and !!!*p* ≤ 0.001). Ca_V,_ voltage-gated Ca^2+^channel; DMSO, dimethyl sulfoxide; I_Ca_, calcium current; RA, retinoic acid.

VF cells were again cultured overnight in the presence or the absence of RA. Each cell was held at −115 mV for 5 s, stepped to −55 mV (approximate RMP) for 5 s, followed by the “control” and then the “modulated” voltage protocols (Fig. 5*A*). We analyzed current area density (total I_Ca_) generated by each spike from both control and modulated voltage protocols and in cells treated with either RA or DMSO (representative traces in Fig. 5*B*). The modulated voltage protocol significantly increased I_Ca_ during each spike (compared with the control protocol), suggesting that the spike broadening and complex spiking induced by 2 μM RA does in fact increase Ca^2+^ influx through Ca_V_ channels. This spike broadening–induced increase in I_Ca_ from the modulated voltage protocol occurred in cells exposed to either RA (*red circles*) or DMSO (*green triangles*) (Fig. 5*C*; # symbols), though was significantly enhanced in DMSO-treated cells compared with RA-treated cells (Fig. 5*C*; spikes 3–9, ∗ symbols). These data therefore suggest that although RA-induced spike broadening significantly enhanced Ca^2+^ influx, this effect was dampened by the concurrent inhibitory effect of RA on Ca_V_ channels.

It is clear that during concurrent spike broadening and exposure to RA, the I_Ca_-enhancing effects of RA predominate. A comparison of I_Ca_ from the modulated protocol in RA-exposed cells (*red circles*), to I_Ca_ from the control protocol in DMSO-exposed cells (*black squares*), indeed produced a significant interaction of treatment and spike number (*F*_(8,162)_ = 4.2; *p* < 0.001) with enhanced I_Ca_ in spikes 2 to 9 of RA-treated cells (Fig. 5*C*; ! symbols). Thus, the inhibition of I_Ca_ (through Ca_V_ channels) by RA limited (but did not prevent) the increase in I_Ca_ that occurred during prolonged depolarizations and neuronal activity (such as during spike broadening and complex spiking).

### RA limits Ca^2+^ influx through Ca_V_2 channels during neuronal activity

Ca_V_1 channels, predominately localized to dendrites and cell bodies (where they play a primary role in activity-dependent gene regulation), appear to be unaffected by RA in physiological Ca^2+^. In contrast, Ca_V_2 channels, predominately localized to presynaptic terminals (where they mediate neurotransmitter release), are inhibited by RA. Therefore, RA-mediated spike broadening and complex spiking, which occur in both cell bodies and isolated neurites (23), may have compartment-specific effects because of different localizations of the Ca^2+^ channel subtypes. We next determined whether the enhanced Ca^2+^ influx occurring during spike modulation occurs *via* Ca_V_1 and/or Ca_V_2 channels.

**Figure 6:**
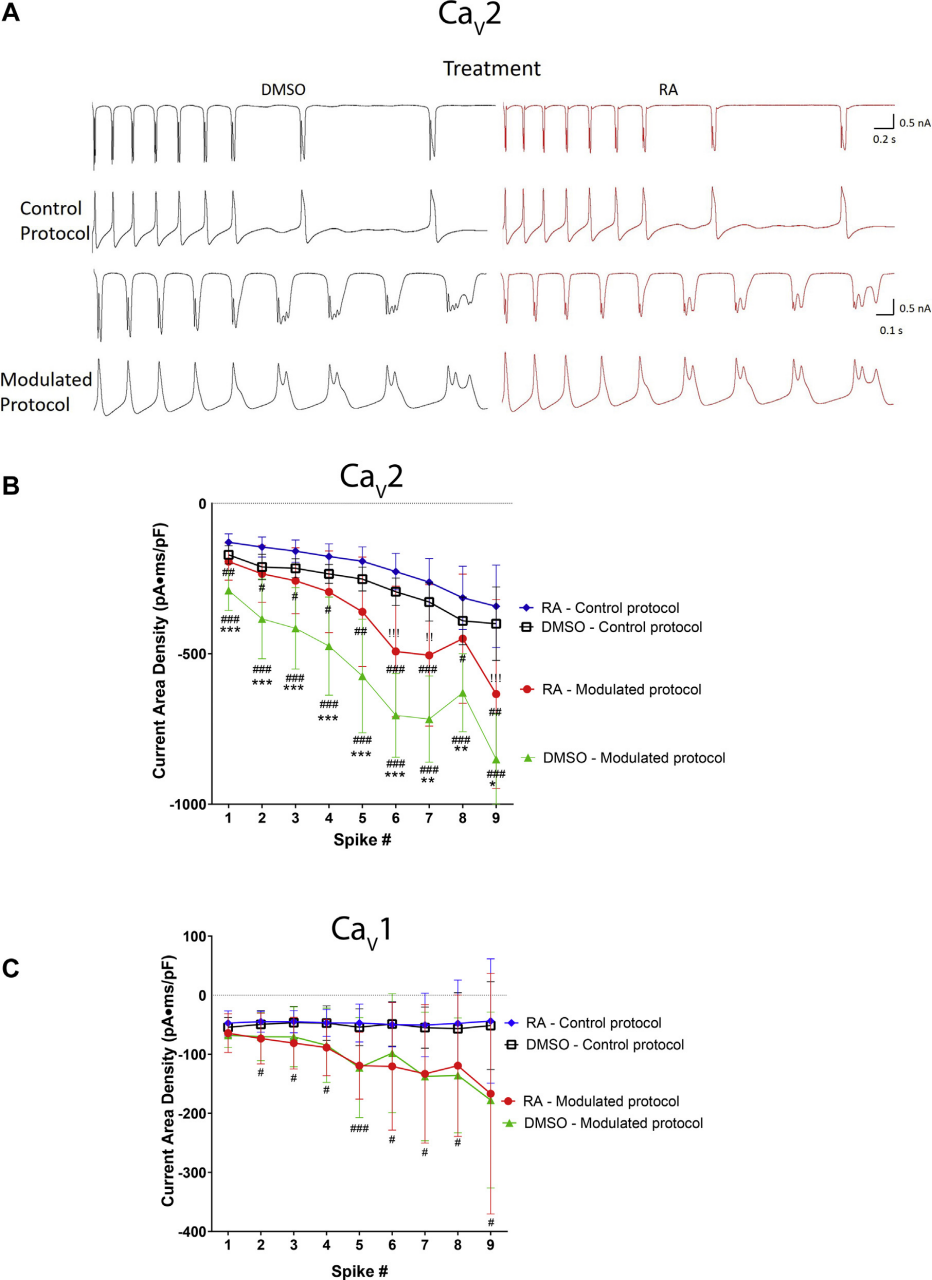
**RA limits Ca**^**2+**^**influx through Ca**_**V**_**2, but not Ca**_**V**_**1, channels during modulated spiking.***A*, raw recordings of Ca_V_2 I_Ca_ (*top traces*) during control and modulated voltage protocols in the presence of 0.02% DMSO (*black*, *left*) or 2 μM RA (*red*, *right*). *B*, graph indicating that the modulated voltage protocol significantly enhanced Ca_V_2 I_Ca_ compared with the control protocol but did so to a greater extent in the presence of DMSO (*green*, n = 11) than in the presence of RA (*red*, n = 10). ∗ represents the comparison between modulated protocols in RA (*red circles*) and DMSO (*green triangles*) (∗*p* ≤ 0.05; ∗∗*p* ≤ 0.01). ! represents the enhanced I_Ca_ between the modulated protocol in RA (*red circles*) and the control protocol in DMSO (*black squares*). !*p* ≤ 0.05; !!*p* ≤ 0.01; !!!*p* ≤ 0.001. *C*, the modulated voltage protocol (in RA and DMSO) significantly enhanced Ca_V_1 I_Ca_ compared with the control voltage protocol. However, there was no significant effect of RA on I_Ca_, compared with DMSO, regardless of voltage protocol. # represents a comparison between modulated and control protocols in both (*B*) and (*C*) (^#^*p* ≤ 0.05; ^##^*p* ≤ 0.01; and ^###^*p* ≤ 0.001). Ca_V_, voltage-gated Ca^2+^channel; DMSO, dimethyl sulfoxide; I_Ca_, calcium current; RA, retinoic acid.

Ca_V_1 and Ca_V_2 currents were isolated and analyzed following application of both control and modulated voltage protocols. In cells exposed to either RA or DMSO, the modulated voltage protocol enhanced I_Ca_ through Ca_V_2 during all spikes, compared with the control voltage protocol (Fig. 6, *A* and *B*; # symbols), confirming that spike broadening and complex spiking enhanced Ca^2+^ influx through Ca_V_2 channels. Once again however, this increase in I_Ca_ through Ca_V_2 was significantly greater following DMSO exposure, compared with RA exposure (Fig. 6*B*; ∗ symbols). These data suggest that spike broadening and activity-dependent complex spiking enhances I_Ca_ through Ca_V_2 and that RA-mediated inhibition of Ca_V_2 limits (but does not prevent) this enhancement. When we again compared Ca_V_2 I_Ca_ following the modulated voltage protocol in cells exposed to RA (*red circles*), with the control voltage protocol in DMSO-exposed cells (*black squares*), there was a significant effect of treatment (two-way ANOVA [*F*_(1,171)_ = 26.156; *p* < 0.001]). However, in this instance, I_Ca_ was only enhanced during the complex spikes (spikes 6, 7, and 9; Fig. 6*B*; ! symbols) but not during the broadened spikes (spikes 1–5).

Analysis of Ca_V_1 currents revealed that the modulated voltage protocol enhanced Ca_V_1 I_Ca_, compared with the control voltage protocol at spikes 2 to 9 (Fig. 6*C*; # symbols) for both RA- and DMSO-treated cells. However, treatment with RA had no significant effect on Ca_V_1 I_Ca_ during the modulated protocols (compared with treatment with DMSO). This suggests that the spike broadening and activity-dependent complex spiking can enhance overall Ca^2+^ signaling through Ca_V_1 channels regardless of concurrent exposure to RA, because unlike Ca_V_2 channels, Ca_V_1 channels are not inhibited by RA in physiological Ca^2+^.

In summary, these data show that activity-dependent spike broadening and complex spiking (such as that induced by RA) results in the enhancement of I_Ca_ through both Ca_V_1 and Ca_V_2 channels. However, exposure to RA also inhibits Ca_V_2 channels (but not Ca_V_1). Though spike broadening is sufficient to compensate for this inhibition, Ca_V_2 I_Ca_ is only enhanced overall during complex spiking and not during spike broadening.

### RA inhibits delayed rectifier K_V_ channels

We have shown that RA inhibits voltage-gated Ca_V_2 channels, but how it affects ion channels to induce spike broadening and complex spiking is not yet known. As K_V_ channels play an essential role in repolarization of the action potential, we next determined whether RA might induce spike broadening by modulating K_V_ function. K_V_ channels include the rapidly inactivating A-type channels and nonrapidly inactivating delayed rectifier channels, known to regulate spike broadening and burst firing in hippocampal neurons, respectively (2, 7). In *Lymnaea* neurons, A-type channels are blocked by 5 mM 4-aminopyridine (4-AP), whereas delayed rectifier channels are blocked by 50 mM tetraethyl ammonium (TEA) (30). In order to isolate A-type channel current (I_A_), total potassium current was first recorded prior to perfusion of 4-AP. Following perfusion of 4-AP, the remaining 4-AP-insensitive current was subtracted from total I_K_, yielding I_A_. Similarly, to isolate the delayed rectifier current (I_KD_) following TEA perfusion, the TEA-insensitive current was subtracted from total I_K_ to yield I_KD_. To determine whether RA affected I_A_ or I_KD_, VF neurons were cultured overnight in the presence or the absence of RA, and the IV relationships for both I_A_ and I_KD_ were established.

**Figure 7:**
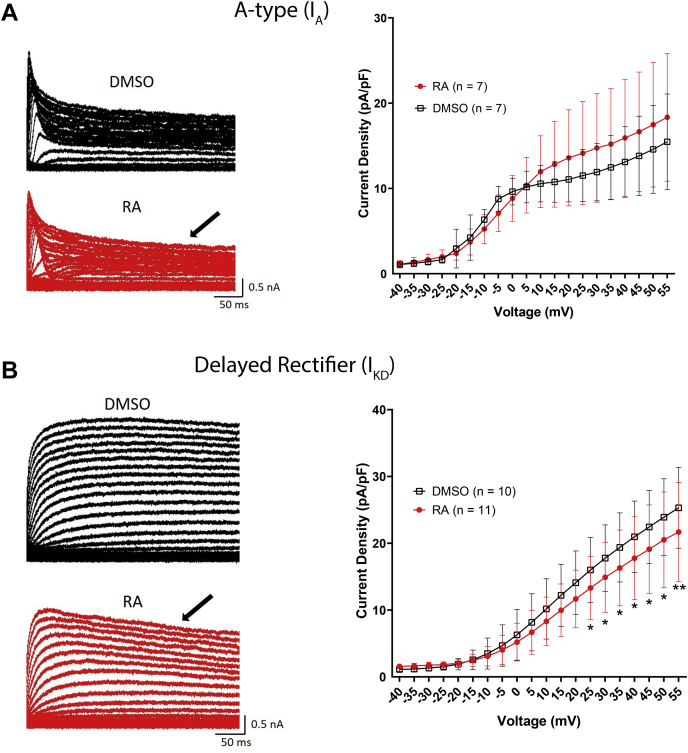
**RA inhibits delayed rectifier K**_**V**_**channels.***A*, raw representative recordings (*left*) of A-type K currents (I_A_) in the presence of 0.02% DMSO (*black*, *top*) or 2 μM RA (*red*, *bottom*). IV relationship (*right*) indicates that RA had no significant effect on the peak current density of A-type channels. *B*, raw recordings (*left*) of delayed rectifier K channels (I_KD_) in the presence of 0.02% DMSO (*black*) or 2 μM RA (*red*). IV relationship (*right*) shows that RA significantly inhibited I_KD_ at potentials between +25 and +55 mV. ∗*p* ≤ 0.05; ∗∗*p* ≤ 0.01, compared with DMSO. *Arrows* on raw traces in (*A*) and (*B*) indicate possible enhanced inactivation in the presence of RA. DMSO, dimethyl sulfoxide; IV, current–voltage; K_V_, voltage-gated K+; RA, retinoic acid.

Raw recordings of I_A_ and I_KD_ following exposure to RA (or DMSO) are shown in Figure 7, *A* and *B*, respectively. A two-way ANOVA of either I_A_ or I_KD_ revealed a significant effect of treatment for I_A_ (*F*_(1,420)_ = 6.059; *p* = 0.014) and I_KD_ (*F*_(1,665)_ = 9.264; *p* = 0.002). However, post hoc analysis revealed nonsignificant effects at all potentials for I_A_, suggesting RA had only minimal effects on I_A_ (Fig. 7*A*). The IV relationship in Figure 7*B* indicates that RA significantly reduced I_KD_ at potentials from +25 to +55 mV, compared with controls (DMSO). As Ca^2+^-activated and Na^+^-activated K^+^ channels might also play a role in action potential repolarization and be affected by RA, we also examined these currents. However, isolation of either Ca^2+^-activated K channels (RA: 1.809 ± 3.0 pA/pF; DMSO: 3.417 ± 4.6 pA/pF) or Na^+^-activated K^+^ channels (RA: 4.227 ± 2.3 pA/pF; DMSO: 2.504 ± 4.0 pA/pF) yielded extremely small peak current densities and were thus not studied further.

Overall, these data suggest that RA inhibits delayed rectifier channels (I_KD_), and that this effect might contribute to the RA-induced spike broadening and activity-dependent complex spiking.

### RA enhances K_V_ channel inactivation

Raw recordings previously shown in Figure 7, *A* and *B* (*arrows*) suggest that RA might enhance the channel inactivation of I_A_ and I_KD_. K_V_ channel inactivation would limit the number of channels available for action potential repolarization and if modulated by RA, might mediate the activity-dependent changes in firing. K_V_ channels can undergo several types of inactivation; A-type channels undergo a rapid form of inactivation, which is coupled to channel opening (referred to as N-type inactivation), as well as a slower form of inactivation, referred to as U-type inactivation (31, 32, 33). Of these two forms of inactivation, delayed rectifier channels might only undergo U-type inactivation, if any (31, 32, 33). This U-type inactivation occurs at intermediate voltages but is reduced at more positive voltages, generating a typical “U” shape in inactivation curves (31, 32, 33).

**Figure 8:**
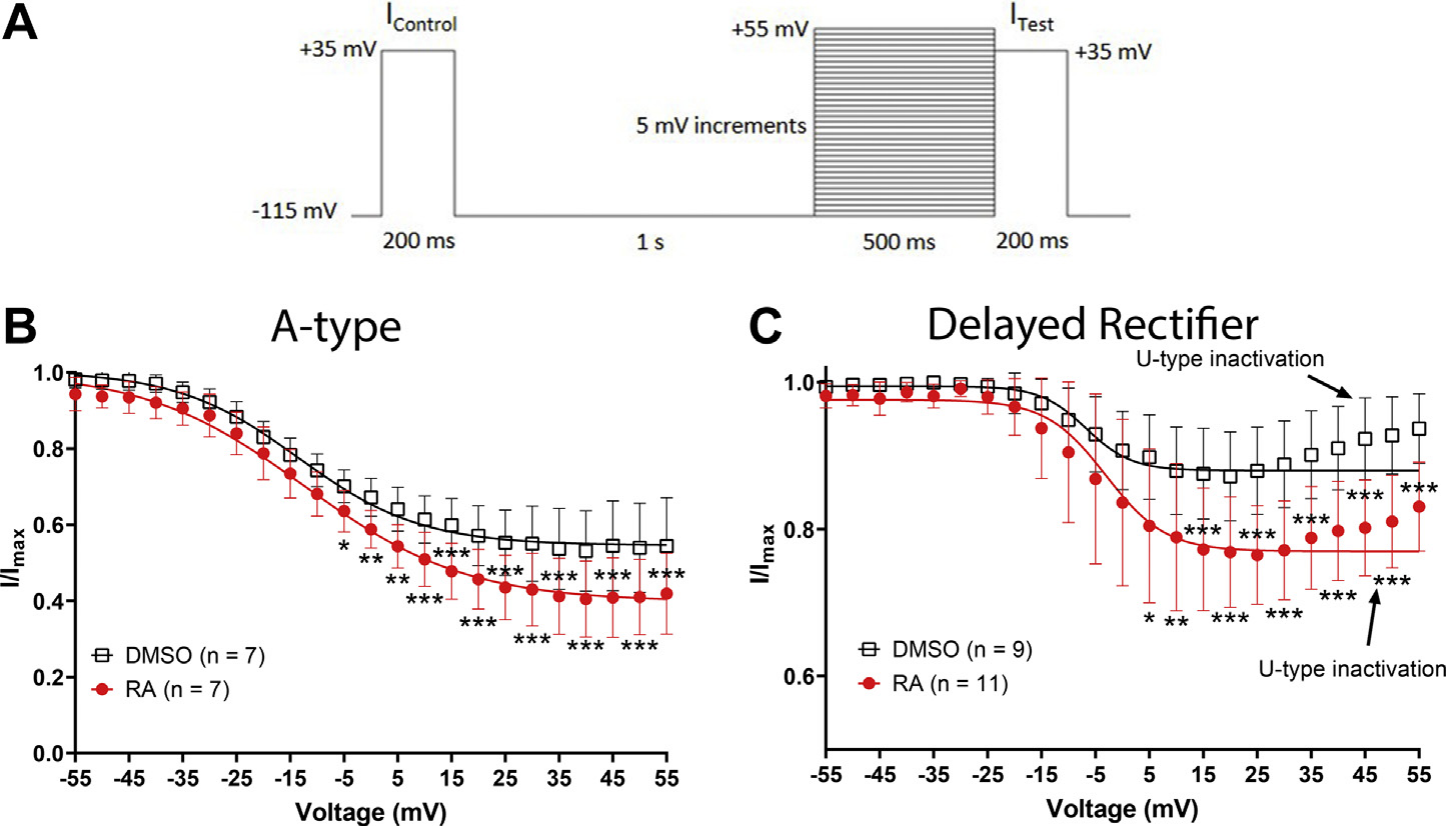
**RA enhances inactivation of A-type and delayed rectifier K**_**V**_**channels.***A*, voltage step protocol used to determine the voltage dependence of inactivation for A-type and delayed rectifier potassium channels. *B* and *C*, inactivation curves for I_A_ and I_KD_ were generated by plotting I/I_max_ (I_test_/I_control_) following steps to different voltages. *Solid lines* represent Boltzmann fits (only between −115 mV and +25 mV for I_KD_ because of U-type inactivation). 2 μM RA significantly enhanced the inactivation of A-type channels (I_A_) at potentials between −5 and +55 mV (*B*) and also enhanced the inactivation of delayed rectifier channels (I_KD_) at potentials between +5 and +55 mV (*C*). *Arrows* in (*C*) indicate the presence of U-type inactivation present in delayed rectifier channels. ∗*p* ≤ 0.05; ∗∗*p* ≤ 0.01; and ∗∗∗*p* ≤ 0.001, compared with DMSO. DMSO, dimethyl sulfoxide; K_V_, voltage-gated K+; RA, retinoic acid.

VF neurons were again cultured overnight in the presence or the absence of RA, and the voltage dependence of channel inactivation of both I_A_ and I_KD_ was examined. Following an initial “control” pulse to +35 mV, cells were stepped from a holding potential of −115 mV to voltages ranging from −115 to +55 mV (for 500 ms) to induce inactivation, followed by a 200 ms “test” pulse to +35 mV (Fig. 8*A*). I_A_ displayed an inactivation curve characteristic of N-type inactivation (Fig. 8*B*), whereas I_KD_ displayed an inactivation curve typical of U-type inactivation (Fig. 8*C*).

A two-way ANOVA of I_A_ inactivation revealed a significant interaction between treatment and voltage (*F*_(34,420)_ = 2.444; *p* < 0.001). Figure 8*B* shows that RA enhanced the inactivation of I_A_ at potentials between −5 and +55 mV, though had no effect on the voltage of half-maximal inactivation (RA: −12.27 ± 7.8 mV; DMSO: −13.393 ± 10.5 mV; *p* = 0.825) or the slope factor (RA: −14.99 ± 2.3; DMSO: −11.38 ± 2.6; *p* = 0.053).

Similarly, a two-way ANOVA of I_KD_ inactivation also revealed a significant interaction between treatment and voltage (*F*_(34,420)_ = 3.689; *p* < 0.001). RA enhanced inactivation of the delayed rectifier channel (I_KD_) at potentials between +5 and +55 mV (Fig. 8*C*) but had no effect on the voltage of half-maximal inactivation (RA: −3.037 ± 9.9 mV; DMSO: −6.753 ± 9.1 mV; *t* = 0.867; *p* = 0.397) but did significantly increase the slope factor (RA: −5.542 ± 1.3; DMSO: −4.258 ± 1.1; *t* = 2.434; *p* = 0.026).

Together, these data suggest that RA enhanced channel inactivation of both A-type (I_A_) and delayed rectifiers (I_KD_) but did not alter the voltage dependence of inactivation for either channel type.

### RA enhances activity-dependent closed state channel inactivation

Recovery from inactivation is also an important property that determines the availability of K_V_ channels for repolarization during neuronal activity. Importantly, K_V_ channels can exist in a closed state (occurring at hyperpolarized potentials and characterized by all four voltage sensors being in the inactive position) or an intermediate-closed state (occurring at intermediate voltages and characterized by at least one of four voltage sensors being in the active position). N-type inactivation subsequently allows for recovery from both closed and intermediate-closed states. U-type inactivation preferentially occurs from intermediate-closed states, and recovery only occurs at potentials that promote the fully closed state (32, 33). This property of U-type inactivation generates a situation whereby repeated opening and closing of the channels results in inactivation, which has been proposed to occur during ongoing neuronal firing (32).

**Figure 9:**
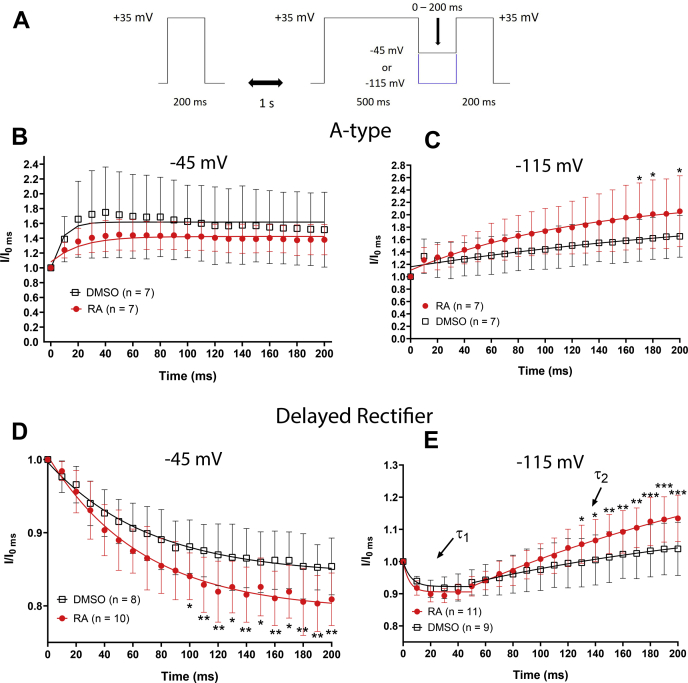
**RA enhances the intermediate-closed state inactivation of delayed rectifier channels.***A*, recovery from inactivation voltage-step protocol for the potassium channel currents, indicating a recovery potential of either −45 mV or −115 mV over variable durations. *B*–*E*, recovery from inactivation curves (for I_A_ and I_KD_), normalized to the level of inactivation at *t* = 0 ms. 2 μM RA had no significant effect on the recovery from inactivation of A-type channels at −45 mV compared with DMSO (*B*) but enhanced the recovery from inactivation of A-type channels at −115 mV, though only at time points between 170 and 200 ms (*C*). Recovery from inactivation of delayed rectifier channels failed to occur at −45 mV (downward curves suggested continued inactivation), which was significantly more pronounced in the presence of RA compared to DMSO (*D*). Because of there being both an inactivation phase and a recovery from inactivation phase at −115 mV (*E*), the recovery from inactivation for the delayed rectifier was fit with two exponentials (τ_1_ τ_2_, *arrows* in *E*). The recovery from inactivation of the delayed rectifier channel was significantly enhanced by RA at −115 mV between 130 ms and 200 ms. ∗*p* ≤ 0.05; ∗∗*p* ≤ 0.01; and ∗∗∗*p* ≤ 0.001, compared with DMSO. DMSO, dimethyl sulfoxide; RA, retinoic acid.

We determined whether RA influences recovery from inactivation at either an intermediate voltage or a hyperpolarized voltage. The protocol used to determine recovery from inactivation is shown in Figure 9*A*. After a control pulse to +35 mV, cells were stepped from a holding potential of −115 mV to +35 mV for 500 ms to induce inactivation and were subsequently stepped down to a potential of either −45 mV (intermediate voltage) or −115 mV (hyperpolarized voltage) for a variable duration between 0 and 200 ms in 10 ms intervals, followed again by a test pulse to +35 mV.

Recovery from inactivation was normalized to the baseline level of inactivation (at *t* = 0 ms) at both −45 mV and −115 mV. A two-way ANOVA for recovery from inactivation of A-type channels (I_A_) revealed a significant effect of treatment at both −45 mV (*F*_(1,252)_ = 19.54; *p* < 0.001) and −115 mV (*F*_(1,252)_ = 34.688; *p* < 0.001). However, the recovery curve at −45 mV was not significantly affected by RA at any particular time point, indicating that RA had minimal effects on recovery from inactivation of I_A_ (Fig. 9*B*). Post hoc analysis of the normalized recovery from inactivation of A-type channels (I_A_) at −115 mV revealed that RA enhanced the recovery from inactivation, compared with DMSO (Fig. 9*C*).

The same analysis was next performed for I_KD._ The recovery from inactivation was again normalized to the baseline level of inactivation (at *t* = 0 ms). Interestingly, at the recovery potential of −45 mV, the current became further reduced over time (Fig. 9*D*), and there was no apparent recovery from inactivation (following treatment with either RA or DMSO). These data strongly suggest that the delayed rectifier channels exhibited intermediate closed-state inactivation at −45 mV. A two-way ANOVA of the normalized recovery from inactivation for I_KD_ revealed a significant effect of RA treatment at −45 mV (*F*_(1,336)_ = 74.261; *p* < 0.001). Specifically, RA significantly reduced I_KD_ at −45 mV (between 100 and 200 ms), suggesting that RA enhanced the intermediate closed-state inactivation at −45 mV (Fig. 9*D*).

In contrast to −45 mV, recovery from inactivation was apparent at the recovery pulse to −115 mV (Fig. 9*E*), indicating voltage dependence in recovery from inactivation. A two-way ANOVA of the normalized recovery from inactivation for I_KD_ revealed a significant interaction between RA treatment and recovery time at −115 mV (*F*_(20,378)_ = 2.622; *p* < 0.001). At −115 mV, RA significantly enhanced recovery from inactivation of I_KD_ at later time points (130–200 ms) (Fig. 9*E*).

In summary, the data obtained at −45 mV (but not −115 mV) suggest that RA enhanced U-type inactivation of I_KD_, coupled to both channel opening and the intermediate-closed state, an effect that is activity dependent. This would likely generate an effect whereby repeated channel open and closure during firing activity in the presence of RA could lead to progressively enhanced inactivation of delayed rectifier channels (I_KD_), potentially leading to activity-dependent complex spiking.

### RA inhibits delayed rectifier channels during neuronal activity

**Figure 10:**
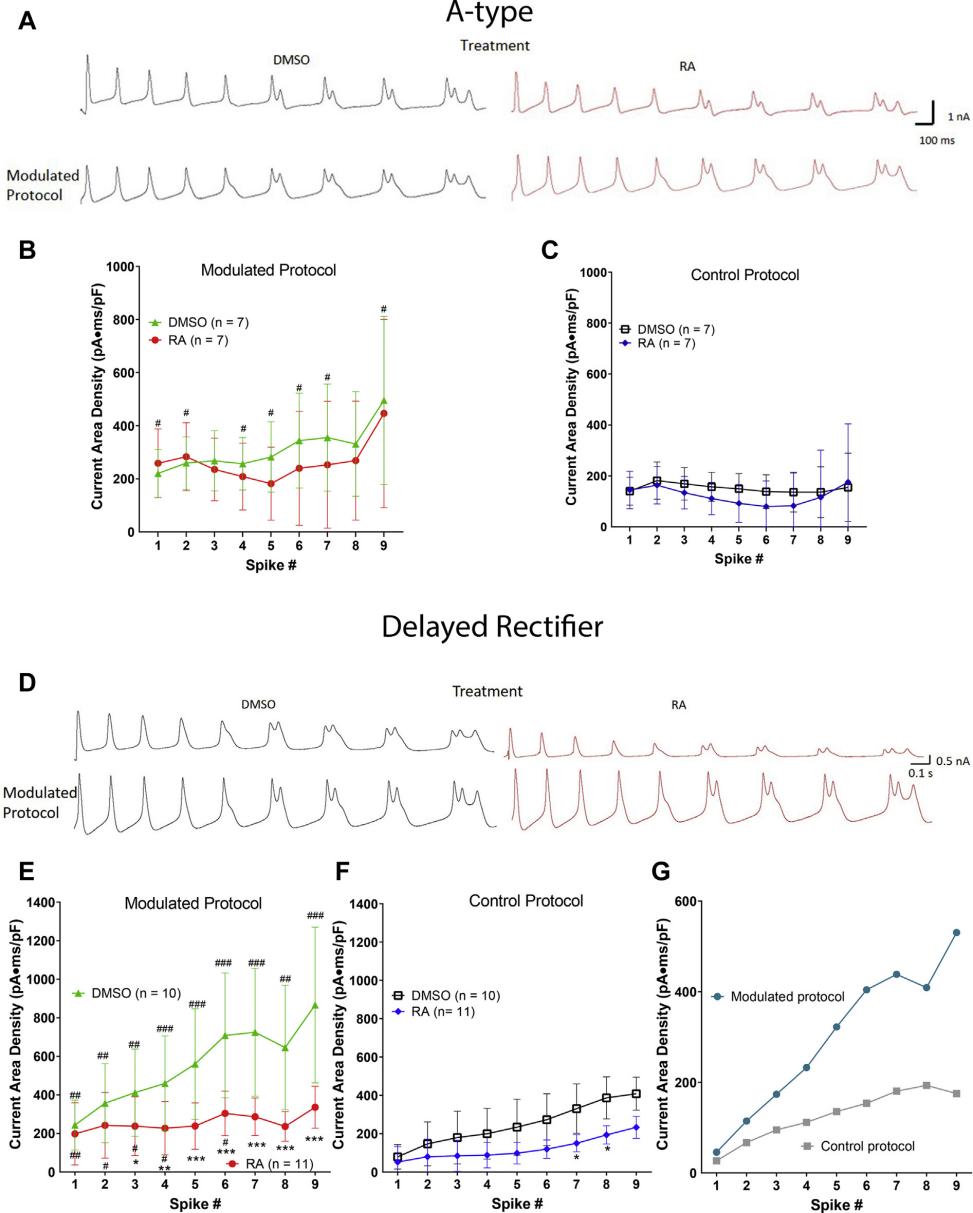
**RA inhibits delayed rectifier channels during neuronal firing.***A*, raw representative recordings of A-type channel currents (I_A_; *top traces*) during modulated voltage protocols (*bottom traces*) in the presence of either 0.02% DMSO (*black*, *left*) or 2 μM RA (*red*, *right*). *B* and *C*, although the modulated protocols exhibited increased current area densities compared with the control protocols, RA had no significant effect on I_A_ compared with DMSO, using either the modulated (*B*) or the control (*C*) voltage protocol. *D*, raw recordings of delayed rectifier channel currents (I_KD_) during the modulated voltage protocol in the presence of either 0.02% DMSO (*black*, *left*) or 2 μM RA (*red*, *right*). *E* and *F*, RA significantly reduced delayed rectifier current area densities during both the modulated voltage protocol (*E*) and the control voltage protocol (*F*). *G*, the average current in RA, subtracted from the average current in DMSO, plotted for both control and modulated voltage, increased as a function of spike number, suggesting that the RA-induced inhibition of the delayed rectifier channel occurs during ongoing neuronal activity and increases with spike number. ∗ represents the comparison between modulated protocols in RA (*red circles*) and DMSO (*green triangles*). ∗*p* ≤ 0.05; ∗∗*p* ≤ 0.01; and ∗∗∗*p* ≤ 0.001. # represents a comparison between modulated and control protocols; ^#^*p* ≤ 0.05; ^##^*p* ≤ 0.01; and ^###^*p* ≤ 0.001. DMSO, dimethyl sulfoxide; RA, retinoic acid.

If, as we suggest previously, the RA-enhanced inactivation of I_KV_ leads to spike broadening and activity-dependent complex spiking, then we predict that exposure to RA would lead to a reduction of I_KD_ during ongoing neuronal activity. To test this hypothesis, we again utilized our control and “modulated” voltage protocols to record either I_A_ or I_KD_ in VF neurons exposed overnight to RA (or DMSO). We first examined whether exposure to RA affected I_A_ during either the modulated (Fig. 10, *A* and *B*) or the control (Fig. 10*C*) voltage protocols. Statistical tests revealed that though the modulated voltage protocol enhanced I_A_ compared with the control protocol (#), there was no significant difference in I_A_ following exposure to RA, compared with DMSO (using either stimulation protocol).

In contrast, we found that exposure to RA significantly reduced I_KD_ during spikes 3 to 9 of the modulated voltage protocol (Fig. 10, *D* and *E*; ∗ symbols). Exposure to RA also reduced I_KD_ during spikes 7 and 8 of the control voltage protocol (Fig. 10*F*; ∗ symbols). These data suggest that exposure to RA inhibited the delayed rectifier channel activity during ongoing neural activity, possibly because of enhanced inactivation as a result of repeated opening and closing of the channel. If this were indeed the case, then the RA-mediated reduction in I_KD_ should be more pronounced with increased neuronal activity and thus, with increasing spike number during the voltage protocols (both control and modulated protocols). Hence, the difference between I_KD_ generated in DMSO and RA for each protocol should also increase with each spike. To examine this, the average I_KD_ in the presence of RA was subtracted from the average I_KD_ in DMSO and plotted for both the modulated and control voltage protocols (Fig. 10*G*). Indeed, the difference in the averaged I_KD_ exhibited an upward trajectory as a function of spike number for both the control protocol (*gray line*) and the modulated spike protocol (*blue line*). These data thus suggest that the RA-induced inhibition of the delayed rectifier channel (but not the A-type channel) occurs during ongoing neuronal activity and increases with spike number.

Overall, we conclude that RA primarily produces activity-dependent modulation of neuronal firing by enhancing U-type inactivation of delayed rectifier channels, which likely occurs as a result of repeated opening and closing of the channel during such ongoing neuronal activity.

## Discussion

In this study, we provide evidence for two neuromodulatory effects of RA that might ultimately affect synaptic transmission. We first show that RA induces complex spiking in an activity-dependent manner (an effect that occurs in multiple cell types) and provide evidence that the underlying mechanism involves enhanced inactivation of delayed rectifier K_V_ channels. However, RA concurrently inhibits Ca_V_2 channels, and we also determined how this interacts with the effects of modulated firing. Our data provide novel evidence to suggest that RA signaling modulates both neuronal firing activity and Ca_V_2 channel gating to tune Ca^2+^ signaling mediated by Ca_V_2 channels.

### RA alters the firing properties of neurons by acting on K_V_ channels

The average concentration of all-*trans* RA in the *Lymnaea* CNS was previously estimated at ∼0.693 μM (26). It is therefore conceivable that local concentrations (in neuronal microdomains) might be comparable to those used here to modulate firing properties and channel activity. We determined that RA concentrations as low as 1 μM induced spike broadening and activity-dependent complex spiking. The acute RA-mediated spike broadening and complex firing in *Lymnaea* neurons was previously shown to be both transcriptionally (23) and translationally independent (34) and did not appear to involve PKA or phospholipase C activation (34). We now provide novel evidence for RA-induced inhibition of neuronal delayed rectifier (K_VD_) channels, which we propose is largely responsible for spike broadening and activity-dependent complex spiking (though the precise molecular signaling pathway by which RA inhibits these channels remains to be determined). To our knowledge, an RA-mediated reduction in delayed rectifier K-channel currents has not previously been shown in the nervous system (in either vertebrates or invertebrates), though has been demonstrated in human lymphocytes (35).

We propose that the RA-induced reduction in delayed rectifier activity might be responsible for the RA-induced spike broadening, as both were found to be independent of activity. However, RA also enhanced inactivation (of both delayed rectifier and A-type channels), and as inactivation is an activity-dependent process, this provides evidence for activity-dependent regulation of both Kv channels by RA. Though we cannot rule out a role for the A-type channel inactivation, we propose that RA likely induces the activity-dependent complex spiking *via* enhanced inactivation of the delayed rectifier (K_VD_) channels. In particular, we show that RA enhanced U-type inactivation, a form of inactivation that occurs at intermediate voltages (corresponding to open and intermediate-closed states). Previous research has shown that delayed rectifier channels expressed in non-neuronal cells (human embryonic kidney cells) exhibit U-type inactivation, and the authors proposed that this may contribute to firing properties during ongoing neuronal activity (32). Repeated opening and closure of the channel during ongoing neuronal activity would result in progressive inactivation because of repeatedly transitioning through “intermediate states.” To our knowledge, this is the first evidence for U-type inactivation of delayed rectifier channels in neurons, and we propose that this U-type inactivation underlies the activity-dependent complex spiking induced by RA.

Activity-dependent complex spiking may be a widespread effect as it occurred in many different cell types (peptidergic and dopaminergic), involved in different behaviors. However, there may be some cell-type specificity, as we show that complex spiking was not activity dependent in the RPA respiratory motor neurons. This differential sensitivity might result from differential expression of either retinoid receptors or ion channels sensitive to RA (and/or the presence or the absence of compensatory mechanisms). The complex spiking was also not activity dependent at higher concentrations, possibly because of greater effects of these higher concentrations on K_V_ channels (35).

Delayed rectifier channels are highly conserved throughout evolution, and both invertebrate- and vertebrate-delayed rectifier channels undergo U-type inactivation when expressed in insect and non-neuronal vertebrate cell lines, respectively (31, 32, 36), suggesting that RA perturbation of these channels and U-type inactivation might modulate neuronal firing activity in both invertebrate and vertebrate neurons. Indeed, inhibiting delayed rectifier channels (such as K_V_2) in rat hippocampal neurons enhances burst firing properties. Interestingly, such burst firing occurs *in vivo* during spatial navigation and learning and memory (7, 24, 37), suggesting a possible mechanism for the known enhancing effects of retinoids on learning and memory in vertebrates (and invertebrates).

### RA signaling regulates activity-dependent neuronal signaling

Both complex spiking and activity-dependent synaptic plasticity are proposed to be the basis for learning and memory. In vertebrates, RA signaling is important for activity-dependent forms of synaptic plasticity, such as LTP and LTD. Indeed, perturbations of retinoid receptors have been shown to affect both synaptic plasticity and behavior; in rodents, downregulation of retinoid receptors (such as retinoic acid receptor β and/or retinoid X receptor γ) disrupts either LTP or LTD and results in impaired novel object recognition and/or impaired working and spatial memories (11, 13, 14, 38).

RA signaling also affects other forms of plasticity in the vertebrate hippocampus, such as homeostatic plasticity, a form of metaplasticity that maintains neuronal activity within an optimal range. Specifically, in response to reduced synaptic activity, RA increases the synthesis (and insertion) of Ca^2+^-permeable α-amino-3-hydroxy-5-methyl-4-isoxazolepropionic acid receptors, an effect that is dependent on NMDA receptor activity (8, 15, 16). Interestingly, this RA-mediated increase in α-amino-3-hydroxy-5-methyl-4-isoxazolepropionic acid receptor expression then blocks subsequent LTP (16), suggesting that the effects of RA on different forms of plasticity are complex, and may have multiple interacting and/or opposing effects. To date, no studies have examined whether RA affects Ca_V_ or K_V_ channel currents in vertebrate neurons and/or synapses.

We have previously shown that RA signaling is also required for normal long-term associative memory formation in *Lymnaea* (21). It is thus feasible that the RA-mediated changes in neuronal firing and Ca^2+^ channel activity might contribute to synaptic plasticity in this molluscan species. Indeed, we have previously shown that inhibiting RA signaling (with retinoid receptor antagonists) produced voltage-dependent inhibition of *Lymnaea* Ca_V_2 channels (29), an ubiquitous form of presynaptic plasticity in vertebrate neurons. The RA-mediated spike broadening shown here is also reminiscent of the spike broadening in presynaptic neurons occurring following behavioral training, and which mediates synaptic facilitation in the mollusc *Aplysia* (39). An important question to consider next is how changes in neural activity (such as during learning) might lead to RA-mediated spike broadening. We propose that RA synthesis might be regulated by neuronal activity. Indeed, RA synthesis is affected by activity at hippocampal synapses (17, 27), and in the *Lymnaea* CNS, we have evidence that expression of the enzyme responsible for RA synthesis increases during memory formation (40).

### Multiple forms of RA-mediated neuromodulation coexist

Multiple forms of RA-mediated modulation might coexist in the same neuron to produce similar outcomes on synaptic plasticity. For example, during homeostatic plasticity in vertebrates, RA signaling enhances excitatory synaptic transmission onto mouse hippocampal neurons whilst independently, but simultaneously, reducing inhibitory synaptic transmission onto the same cells by enhancing endocytosis of the postsynaptic gamma-aminobutyric acid receptors. The coexistence of these differing effects of RA leads to a shift in synaptic weight to produce an overall increase in excitability (18).

Our study has now also provided evidence for multiple modes of RA-mediated modulation coexisting and interacting in invertebrate neurons, though in this case, the effects oppose each other. We show that a concentration of RA (2 μM) that produces activity-dependent complex spiking (enhancing Ca^2+^ influx) also inhibits Ca^2+^ influx through Ca_V_2 channels. We further provide evidence that these differing effects of RA interact. By using playback of modulated spiking protocols in the presence or the absence of RA, we found that RA-mediated inhibition of Ca_V_ channels limits the enhanced Ca^2+^ influx that occurs during RA-mediated spike broadening and complex spiking. However, when the Ca_V_2 channel currents were examined in isolation, the inhibition of Ca_V_2 by RA prevented the increase in Ca^2+^ influx during spike broadening. Indeed, it was only during complex spiking that the enhanced influx of Ca^2+^ dominated and overcame Ca_V_2 channel inhibition.

Despite these multimodal modulatory effects of RA, it is unlikely that the RA-mediated inhibition of Ca_V_2 channels is directly responsible for the RA-mediated changes in neuronal firing. Ca_V_ channels were blocked whilst examining actions of RA on I_KD_, and thus changes in Ca^2+^ influx (through Cav2s) could not account for these results. Furthermore, reduced Ca^2+^ influx is unlikely to affect Ca^2+^-activated K channels, as these particular channels generate very little current in these cells. RA-mediated effects on spiking might, however, have led to a homeostatic reduction in Ca_V_2 activity (or vice versa), though we consider this unlikely as both effects of RA (on spiking and Ca_V_ channels) can occur rapidly and over similar timescales (34). We thus propose that although RA-mediated modulation of spike activity and inhibition of Ca_V_2 channels coexist in the same cell type, that they are likely independent effects.

### Differential effects of RA on Ca_V_1s *versus* Ca_V_2s

We determined that spike broadening and complex spiking enhanced Ca^2+^ influx through Ca_V_1 channels, though simultaneous RA exposure did not limit this, because RA did not inhibit Ca^2+^ influx *via* Ca_V_1. Interestingly, we previously found that higher concentrations of RA (5 μM) did inhibit Ca_V_1 channels, though using barium instead of physiological levels of Ca^2+^ (25). This may have affected the outcome, if we consider the possibility that the presence of Ca^2+^ might influence the actions of RA on Ca_V_1 channels.

Because of the different locations and roles of Ca_V_1 and Ca_V_2 channels in neuronal microdomains, our results suggest a scenario whereby RA-induced modulation of spiking might enhance Ca^2+^ signaling involved in gene regulation (Ca_V_1s) but would limit the enhancement in Ca_V_2-mediated synaptic signaling. Moreover, Ca_V_1 channels have been implicated in presynaptic plasticity mechanisms, such as presynaptic LTP (41), as well as neuropeptide release (42), suggesting that specific regulation of Ca_V_2 (but not Ca_V_1) channels by RA during activity-dependent changes in spiking may ultimately target selective Ca^2+^ signaling pathways or functions. RA-mediated modulation of neuronal activity and its differential modulation of Ca_V_1 and Ca_V_2 channels may thus be a mechanism to fine-tune information processing in different compartments in invertebrate or vertebrate neurons.

### Possible consequences for synaptic transmission and plasticity

In both vertebrate and invertebrate neurons, depolarization during action potential firing activates Ca_V_2 channels present in presynaptic terminals, with the resulting Ca^2+^ influx mediating neurotransmitter release. When the duration of depolarization is prolonged, during either spike broadening or complex spiking, it would likely increase the fidelity and magnitude of synaptic transmission. Indeed, we found that RA-mediated complex spiking enhanced Ca_V_2-mediated I_Ca_, though RA-mediated inhibition of Ca_V_2 channels compensated for any increase in I_Ca_ during spike broadening. As such, the system might act as a high-pass filter, whereby only activity leading to complex spiking enhances synaptic output. The ability of RA to limit enhancement of I_Ca_ (*via* inhibition of Ca_V_2s) might also prevent synaptic “runaway.” Indeed, it has been shown that RA signaling can limit LTP in mice following environmental enrichment (43), and if RA signaling is disrupted, mice exhibit synaptic runaway, resulting in reduced cognitive flexibility. It should also be noted that during playback of the normal control-spiking protocols, RA also induced a reduction in Ca_V_2 currents, which, albeit small and nonsignificant, might also have physiological consequences on synaptic transmission. It is thus possible that RA might also inhibit Ca_V_2-mediated synaptic transmission during “normal” spike trains.

In this study, we present the first cellular evidence for activity-dependent modulation by RA in an invertebrate CNS. However, whether the RA-mediated activity-dependent complex spiking plays a direct role in synaptic plasticity and/or memory formation requires further evaluation. In mouse hippocampal neurons, complex spiking, such as burst firing of presynaptic neurons, is involved in memory formation (24). In contrast, burst firing in postsynaptic neurons can induce homeostatic synaptic depression (44), and postsynaptic spike broadening can lead to LTD (45). Thus, determining the exact role of RA-mediated spike broadening and activity-dependent burst firing during synaptic transmission, by examining RAs effects in both presynaptic and postsynaptic cells, will be needed.

It is very likely that both short-term and long-term consequences of complex spiking on synaptic transmission will be synapse specific and might depend on the release properties and dynamics of that particular synapse. Furthermore, the increases in Ca^2+^ influx during complex spiking will likely modulate various kinase pathways and/or local protein synthesis, as well as potentially mediate neuropeptide or neurotrophin release, in addition to the aforementioned gene transcription. To add a further level of complexity, RA is known to enhance neurotransmitter release from *Xenopus* motor neurons, in a manner that is independent of extracellular calcium (46). Thus, determining the precise role of RA-induced activity-dependent complex spiking in synaptic modulation and plasticity will require a thorough study of a number of different synapses, utilizing different neurotransmitters.

In summary, the ability of RA to inhibit Ca_V_2 channels must coexist with its ability to induce spike broadening and activity-dependent complex spiking, which ultimately enhances Ca^2+^ influx. This concurrent inhibition of Ca_V_2 channels thus influences and fine-tunes Ca^2+^ signaling during ongoing neuronal activity.

## Experimental procedures

### Animals

The gastropod mollusc, *L. stagnalis*, utilized in these experiments was bred at Brock University and maintained at room temperature on a fixed 12:12 h light–dark cycle in aerated dechlorinated water. Animals were fed a combination of romaine lettuce and Nutrafin Max Spirulina fish food (Hagen) daily. CNS from adult animals (20–23 mm in shell length) were used for cell culture.

### Cell culture

All dissections were performed under sterile conditions as previously described (25, 29). Briefly, *Lymnaea* were anesthetized through incubation in saline containing 25% Listerine (containing menthol, 0.042% w/v). *Lymnaea* were subsequently pinned in a dissection dish containing antibiotic saline (normal saline containing 225 μg/ml gentamicin [Sigma–Aldrich]) and dissected to expose the CNS consisting of the central ring ganglia, which was removed and given three 10 min washes in antibiotic saline. The CNS was treated with trypsin (Sigma–Aldrich; 2 mg/ml) in defined medium (DM; 50% Leibovitz’s L-15 medium; Gibco) for 18 to 19 min at 21 °C and subsequently treated with trypsin inhibitor (Sigma–Aldrich; 2 mg/ml in DM) for 10 min at 21 °C. The CNSs were then pinned out in high-osmolarity DM, followed by removal of the outer sheath of connective tissue. The inner sheath was subsequently removed to expose the neurons. Individually identified neurons were removed from the ganglia using a fire-polished glass pipette coated with Sigmacote (Sigma–Aldrich) to prevent cell adhesion. Gentle suction was applied *via* a microsyringe (Gilmont) to remove individual neuronal cell bodies from the CNS. Individual neurons were subsequently plated in DM on plastic culture dishes coated with poly-l-lysine (Sigma–Aldrich). Cells were then treated with all-*trans* RA (Sigma–Aldrich), prepared as a 10^−2^ M stock solution in 100% DMSO, and diluted in DM to produce a final bath concentration ranging from 1 to 5 μM. Control cells were treated with an equivalent concentration of DMSO. The different cell types used in this study included VF peptidergic neurons, the dopaminergic RPeD1, RPB, and RPA cells. VF neurons were the predominant cell type used. Between five and eight VF cells were generally isolated from one CNS and plated in either one or two culture dishes. Most experiments used only one cell/dish (unless stated otherwise), and so no more than 1 to 2 cells from each CNS were utilized for recordings.

The firing properties of cultured neurons were assessed using current-clamp electrophysiology, whereas the biophysical properties of Ca_V_ and K_V_ channels were assessed using whole-cell voltage clamp electrophysiology (see later for details). All recordings from cultured cells were performed at room temperature, using a MultiClamp 700A amplifier, a Digidata 1322A digitizer, and Clampex 9.2 software (Molecular Devices). All external solutions were designed to mimic the ionic constituents of DM and to maintain a liquid junction potential of approximately +15 mV between all solutions, which was adjusted for.

### Current clamp

Current-clamp recordings were performed in DM. Patch pipettes with a resistance between 2 and 6 MΩ were filled with an internal solution containing 2 mM Mg-ATP, 0.1 mM GTP–Tris, 5 mM EGTA, 1 mM CaCl_2_, 10 mM Hepes, 60 mM potassium gluconate with a pH of 7.4 achieved using potassium hydroxide (30). To stimulate action potential firing, cells were held at 0 pA for 4 s followed by current steps between −100 and +400 pA in 25 pA intervals for 5 s. Current-clamp recordings were not limited to one cell per dish, but only cells that had an RMP more negative than −40 mV were used. Recordings were performed on cells with an access resistance of <15 MΩ and performed at room temperature. Because of the variability in capacitance between VF neurons (100–300 pF) and between the four different cell types used in this study (100–600 pF), the firing properties of cells were assessed at the first current step in which at least three action potentials were elicited (rheobase 1) as well as the three subsequent current steps (referred to as rheobases 2–4). To assess spike broadening, the half-width of the first three spikes in a particular current step was averaged, as done previously (23).

To confirm there was an increase in the number of spikes (events) across rheobases 1 to 4, the number of spikes was counted for each rheobase (independent of whether it was a complex spike) and statistically analyzed to confirm an increase in activity across rheobases. To then assess activity-dependent complex spiking, the number of complex spikes was counted at rheobases 1 to 4. A cell was deemed to exhibit complex spiking if a doublet, triplet, burst, or plateau potential occurred at any of the four rheobases.

Additional electrophysiological parameters measured included the voltage at which the peak of the action potential occurred, the peak voltage of the AHP, RMP, and input resistance. The equation, $R=\frac{\tau \;}{C}\;$, was used to calculate the input resistance (obtained from hyperpolarizing current pulses) in each cell.

### Ca_V_ channel recordings

I_Ca_ for both voltage-clamp and action potential clamp was assessed using an external solution containing 4.1 mM CaCl, 40 mM tetramethylammonium chloride, 1.5 mM MgCl_2_, 10 mM Hepes, and 5 mM 4-AP, at a pH of 7.9, achieved using TEA-OH. Patch electrodes with a resistance between 2 and 6 MΩ were filled with internal solution containing 29 mM CsCl, 1 mM CaCl_2_, 2 mM MgATP, 0.1 mM GTP–Tris, 5 mM EGTA, 10 mM Hepes, and 60 mM potassium gluconate with a pH of 7.4 achieved using cesium hydroxide. Recordings were performed on cells with an access resistance of <15 MΩ with series resistance compensation to 85%. As spike firing characteristics could not be recorded in the exact same cell as I_Ca_, in order to ensure that the RA-mediated effects were occurring in parallel, two VF cells from the same CNS were consistently plated together in one dish and exposed to identical treatments. One cell was first used to confirm the RA-mediated effects on spike broadening (current clamp), whereas the second cell was then used to record RA-mediated effects on I_Ca_ (voltage clamp).

### Characterization of Ca_V_ channel subtype using nifedipine

The selective L-type Ca^2+^ channel blocker, nifedipine, was used to determine the effects of RA on L-type *versus* non–L-type Ca_V_ channels. Cells were incubated overnight in either 2 μM RA or 0.02% DMSO (controls). A stock solution of nifedipine was prepared at a concentration of 10^−2^ M using 100% DMSO. Neurons that had been exposed to either RA or DMSO were first acutely perfused with external solution containing 0.1% DMSO (as a vehicle control), and an IV relationship was obtained in DMSO, which represented total I_Ca_. This solution was then replaced with external solution containing 10 μM nifedipine.

VF neurons were held at −115 mV and stepped to +5 mV for 200 ms once every 30 s while the inhibitor, nifedipine, was applied to the bath, until peak inhibition was attained. The IV relationship of the remaining nifedipine-insensitive I_Ca_ through Ca_V_2 (non–L-type) was established. The nifedipine-sensitive I_Ca_ through Ca_V_1 (L type) was then determined by subtracting nifedipine-insensitive I_Ca_ from total I_Ca_. Subtraction was performed offline using Clampfit 9.2 (Molecular Devices). The voltage dependence of channel activation and the voltage of half-maximal activation were determined as described later. To assess the amount of channel rundown that might occur during nifedipine-mediated L-type Ca^2+^ channel block, additional control experiments were performed in which cells were only exposed to 0.1% DMSO, and rundown was assessed over a similar period over which nifedipine block would normally occur. Voltage steps to +5 mV (from a holding potential of −115 mV) were conducted every 30 s over a time course of 10 min, and revealed no subsequent rundown of I_Ca_ (an actual 4.4% increase in I_Ca_ occurred; n = 6).

### K_v_ recordings

K_V_ channel currents (I_K_) were recorded using the same internal solution as used for current-clamp recordings. Prior to obtaining recordings of K^+^ currents in voltage-clamp mode, voltage recordings of the firing properties of VF neurons (treated with either RA or DMSO) were obtained in current-clamp mode. This was done in the presence of DM using a modified version of the current-clamp protocol described previously, to ensure that RA consistently produced spike broadening and complex spiking. The DM was then replaced with the appropriate K^+^ channel external solution, depending on the K^+^ channel type being isolated. In this study, we isolated four different types of K^+^ channels: A-type K_V_ channels, delayed rectifier K_V_ channels, Na-activated K^+^ channels, and Ca^2+^-activated K^+^ channels. To isolate Na-activated K^+^ channels, K^+^ currents were first recorded in an external solution containing 40 mM NaCl, 1.7 mM KCl, 4.1 mM CaCl_2_, 1.5 mM MgCl_2_, 10 mM Hepes, and pH 7.9 with potassium hydroxide (Na^+^-containing external solution). This external solution was then replaced with an external solution where NaCl has been substituted with the Na^+^-channel impermeate ion, tetramethylammonium (40 mM tetramethylammonium chloride; Na^+^-free external solution). Na^+^-activated K^+^ channel currents were obtained by subtracting K^+^ currents obtained in Na^+^-free external solution from K^+^ currents obtained in Na^+^-containing external solution. Ca^2+^-activated K^+^ channel currents were obtained by first recording I_K_ in Na^+^-free external solution (Total I_K_). This Na^+^-free external solution was then replaced with Na^+^-free external solution containing 30 μM cadmium to block I_Ca_, and I_K_ was subsequently established. Subtraction of I_K_ remaining in the presence of cadmium from total I_K_ yielded Ca^2+^-activated K^+^ currents.

Isolation of A-type I_K_ was achieved using 4-AP. I_K_ was first recorded in Na^+^-free external solution (total I_K_). This Na^+^-free external solution was then replaced with Na^+^-free external solution containing 5 mM 4-AP, and I_K_ was again established. Subtraction of I_K_ remaining in the presence of 4-AP from total I_K_ yielded A-type I_K_. Isolation of delayed rectifier (I_KD_) was achieved using TEA. Total I_K_ was first recorded in Na^+^-free external solution containing 30 μM cadmium as calcium-activated K^+^ channels are also inhibited by TEA. This external solution was then replaced with Na^+^-free external solution containing 30 μM cadmium and 50 mM TEA, and I_K_ was again established. Subtraction of I_K_ remaining in the presence of TEA from total I_K_ yielded delayed rectifier I_KD_.

### IV relationship

To determine the IV relationship for I_Ca_ and I_K_, cells were held at −115 mV and stepped to membrane potentials ranging from −115 to +55 mV in 5 mV increments for 400 ms and stepped down to −115 mV for 1 s to remove any inactivation. Peak I_Ca_ or I_K_ was measured for each voltage step using Clampfit 9.2 and normalized to cell capacitance. For total and Ca_V_2 I_Ca_, an ohmic leak conductance was present. This was calculated from a +10 mV step and subtracted offline. The IV relationships were compared across treatment conditions using a two-way ANOVA, followed by a Bonferroni post hoc test.

### Voltage dependence of channel activation

To assess the voltage dependence of Ca^2+^ channel activation, channel conductance was first calculated for each membrane potential used in the IV relationship protocol described previously. Conductance was calculated using the equation, $\frac{I}{\left(Vm-Erev\right)}$, where *I* represents current, *Vm* represents membrane potential, and *Erev* represents the reversal potential. *Erev* was calculated by linear extrapolation of the IV curve at potentials more depolarized than that at which the peak current occurs. Conductance values for each voltage were normalized to the maximal conductance, *g*_max_, for each neuron. The values of *g*/*g*_max_ were fit to the Boltzmann equation:

f(V)=gmax1+eVmid−VVc+C

where *Vmid* represents the voltage of half-maximal activation, *V* represents the membrane potential, *Vc* represents the slope factor, and *C* represents a constant. The voltage of half-maximal activation and slope factor produced for each treatment condition were compared using an unpaired *t* test or a Mann–Whitney rank sum test, as appropriate.

### Voltage dependence of channel inactivation

To determine the voltage dependence of Ca_V_ and Kv channel inactivation, cells were held at −115 mV and stepped to +5 mV for I_Ca_ or +35 mV for I_K_ for 200 ms to generate the control I_Ca_ or I_K_ (I_control_). I_control_ was measured with each sweep of the protocol to account for any rundown that may have occurred over the course of the recording. Voltage-dependent inactivation was achieved by holding cells at −115 mV and stepping to membrane potentials between −115 and +55 mV for 500 ms in 5 mV increments. The membrane potential was then immediately stepped to +5 mV for I_Ca_ or +35 mV for I_K_ for 200 ms to generate the test current (I_test_) (25). To generate a steady-state inactivation curve, I_test_/I_control_ was calculated for each test potential. Values for each test potential were then fit in Clampfit using the Boltzmann equation. In the case of delayed rectifier channels, as a result of U-type inactivation, Boltzmann fits were only performed on voltages between −115 and +25 mV, which is where the inactivation peaks, but this fit line was extended to +55 mV in Figure 8*C* to illustrate the U-type inactivation. The voltage of half-maximal inactivation and the slope factor produced during each treatment condition were compared using an unpaired *t* test or a Mann–Whitney rank sum test, as appropriate.

### Recovery from channel inactivation

In order to determine the recovery from inactivation, cells were held at −115 mV and stepped to +5 mV for I_Ca_ or +35 mV for I_K_ for 200 ms to generate I_control_. Voltage-dependent inactivation was achieved by depolarizing the membrane potential to +5 mV for I_Ca_ or +35 mV for I_K_ for 500 ms. The membrane potential was then returned to −115 mV for I_Ca_ (or to both −115 mV and −45 mV for I_K_) for a variable duration of time ranging from 0 to 1 s in 50 ms intervals for I_Ca_ or 0 to 200 ms in 10 ms intervals for I_K_, to allow the channels to recover from inactivation. The membrane potential was then stepped to +5 mV for I_Ca_ or +35 mV for I_K_ for 200 ms to generate the test current (I_test_). I_test_ produced during each recovery period was then normalized to I_control_ and plotted against the duration of time allowed for recovery. The resultant recovery from inactivation curve was compared across treatment conditions using a two-way ANOVA. To determine the time constant, the recovery from inactivation curve was fit with the exponential equation. The time constant of the recovery from inactivation was compared between treatment groups using an unpaired *t* test or a Mann–Whitney rank sum test.

### Action potential clamp

To determine whether the RA-mediated spike broadening and complex spiking influenced Ca_V_ and K_V_ channels, we utilized whole-cell action potential clamp. Representative current-clamp voltage traces at rheobase 4 of a cell pretreated with either 2 μM RA (“modulated” protocol) or 0.02% DMSO (“control” protocol) were used as the voltage stimulus. VF neurons were exposed to either 2 μM RA or 0.02% DMSO overnight, whole-cell voltage clamped, and exposed to both the “control” protocol and the “modulated” voltage protocols. Both the control and modulated protocols consisted of nine spikes. Cells were held at −115 mV for 5 s to remove any inactivation and then stepped to −55 mV for 5 s to allow ion channels to occupy the appropriate gating states at the approximate RMP. Cells were then stimulated with the control and modulated voltage protocols. To remove the noise in the current recording that was generated as a result of the noise in the voltage recordings (used as the voltage protocols), a 200 Hz low-pass filter was applied to each recording offline in Clampfit. Each recording was manually inspected before and after this filter was applied to ensure only noise was removed. The current area that was coincident with each spike in both protocols was quantified in Clampfit and normalized to the capacitance of each cell, and the current remaining at the end of the action potential firing protocol was subtracted as leak current.

Four experimental conditions were analyzed: (1) overnight exposure to 2 μM RA, followed by the control protocol; (2) overnight exposure to 2 μM RA, followed by the modulated voltage protocol; (3) overnight exposure to 0.02% DMSO, followed by the control protocol; and (4) overnight exposure to 0.02% DMSO, followed by the modulated voltage protocol. Groups were compared across all nine spikes (of the protocol) using a two-way ANOVA. To determine the contribution of capacitative current (during total I_Ca_ and Ca_V_2 I_Ca_) during action potential clamp, control experiments were performed in Ca^2+^ channel external solution containing 0 mM Ca^2+^ and 30 μM cadmium to block all Ca^2+^ influx and revealed that the capacitative current was negligible.

### Statistical analysis

All statistical analyses were performed using SigmaStat 3.2 (Sysstat), and graphs were generated using GraphPad Prism 5.03 (GraphPad Software, Inc). Values are presented as mean ± SD, and differences were deemed significant when *p* ≤ 0.05.

## Data availability

All data are contained within the article.

## Conflict of interest

The authors declare that they have no conflicts of interest with the contents of this article.
